# LncRNA AC006064.4–201 serves as a novel molecular marker in alleviating cartilage senescence and protecting against osteoarthritis by destabilizing CDKN1B mRNA via interacting with PTBP1

**DOI:** 10.1186/s40364-023-00477-6

**Published:** 2023-04-13

**Authors:** Panyang Shen, Jun Gao, Shaohan Huang, Chenan You, Haitao Wang, Pengyu Chen, Teng Yao, Tianyou Gao, Bohao Zhou, Shuying Shen, Xing Zhao, Jianjun Ma

**Affiliations:** 1 Department of Orthopaedic Surgery, Sir Run Run Shaw Hospital, Medical College of Zhejiang University & Key Laboratory of Musculoskeletal System Degeneration and Regeneration Translational Research of Zhejiang Province, 3 East Qingchun Road, Hangzhou, 310016 Zhejiang Province China; 2 Department of Endocrinology, Sir Run Run Shaw Hospital, Medical College of Zhejiang University, 3 East Qingchun Road, Hangzhou, 310016 Zhejiang Province China

**Keywords:** lncRNAs, Osteoarthritis, Senescence, PTBP1, CDKN1B

## Abstract

**Background:**

Osteoarthritis (OA) is the most prevalent age-related disease in the world. Chondrocytes undergo an age-dependent decline in their proliferation and synthetic capacity, which is the main cause of OA development. However, the intrinsic mechanism of chondrocyte senescence is still unclear. This study aimed to investigate the role of a novel long non-coding RNA (lncRNA), AC006064.4–201 in the regulation of chondrocyte senescence and OA progression and to elucidate the underlying molecular mechanisms.

**Methods:**

The function of AC006064.4–201 in chondrocytes was assessed using western blotting, quantitative real-time polymerase chain reaction (qRT-PCR), immunofluorescence (IF) and β-galactosidase staining. The interaction between AC006064.4–201 and polypyrimidine tract-binding protein 1 (PTBP1), as well as cyclin-dependent kinase inhibitor 1B (CDKN1B), was evaluated using RPD-MS, fluorescence in situ hybridization (FISH), RNA immunoprecipitation (RIP) and RNA pull-down assays. Mice models were used to investigate the role of AC006064.4–201 in post-traumatic and age-related OA in vivo.

**Results:**

Our research revealed that AC006064.4–201 was downregulated in senescent and degenerated human cartilage, which could alleviate senescence and regulate metabolism in chondrocytes. Mechanically, AC006064.4–201 directly interacts with PTBP1 and blocks the binding between PTBP1 and CDKN1B mRNA, thereby destabilizing CDKN1B mRNA and decreasing the translation of CDKN1B. The in vivo experiments were consistent with the results of the in vitro experiments.

**Conclusions:**

The AC006064.4–201/PTBP1/CDKN1B axis plays an important role in OA development and provides new molecular markers for the early diagnosis and treatment of OA in the future.

**Graphical Abstract:**

Schematic diagram of AC006064.4–201 mechanism. A schematic diagram of the mechanism underlying the effect of AC006064.4–201
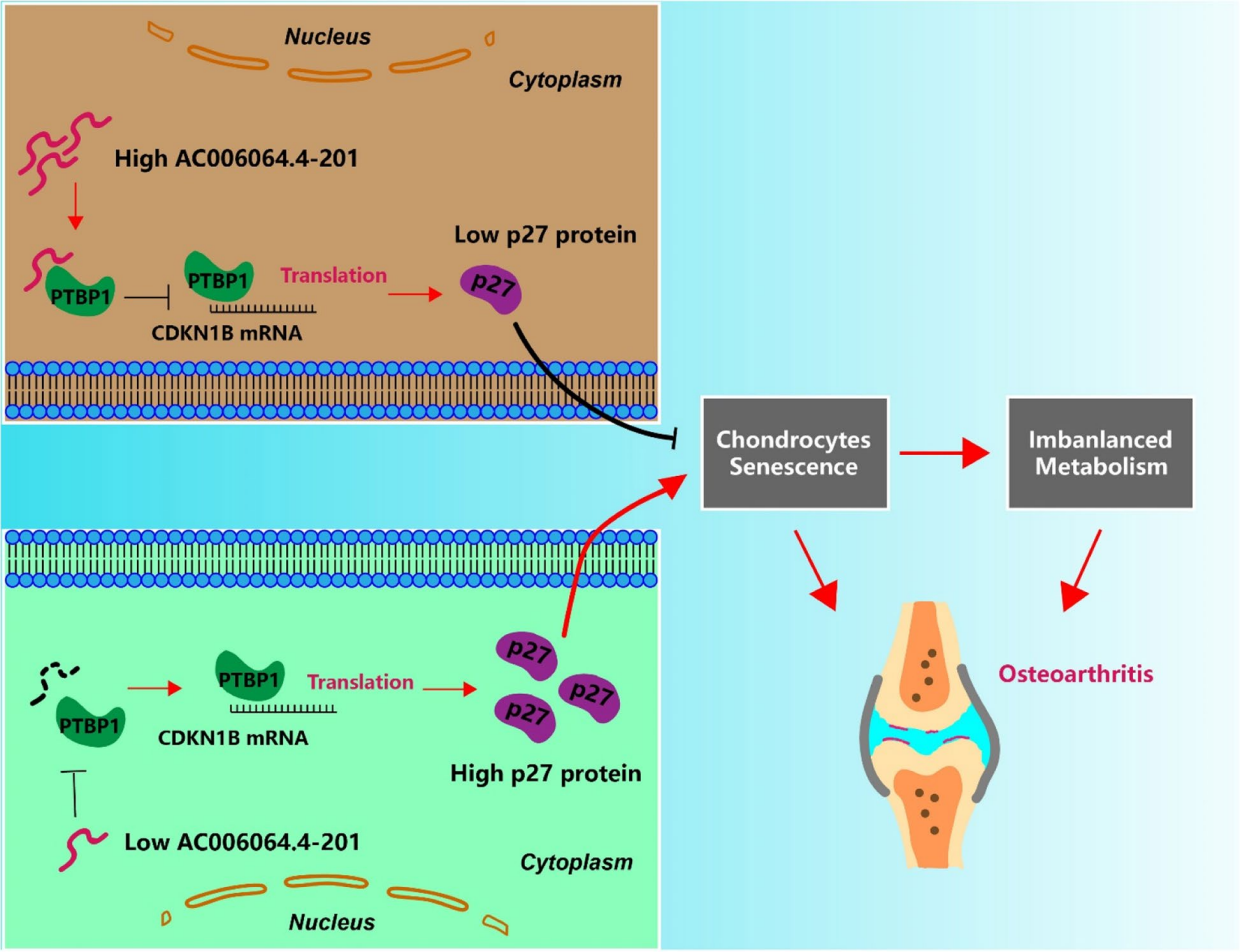

**Supplementary Information:**

The online version contains supplementary material available at 10.1186/s40364-023-00477-6.

## Introduction

OA is the most prevalent degenerative disease and the main cause of disability in older adults worldwide. It has affected 32.5 million adults in the United States, at an annual cost of $136.8 billion a year [1, 2]. There are many risk factors for OA, including aging, sex, obesity, genetics, early joint trauma, and muscle weakness, of which the most important is aging [3, 4]. With the extension of human life, the morbidity of OA will gradually increase. It is predicted that more than 67 million people in the United States will suffer from OA by 2030 and exceed 78.4 million by 2040 [5, 6]. However, pathogenesis of OA remains unclear.

Cellular senescence is a stress response induced by multiple intrinsic and extrinsic stimulators [7]. It is currently one of the most rapidly developing branches of science and has been directly implicated as a key driver of age-related diseases [8, 9]. Secondary senescence and aggravated tissue damage are caused by the local accumulation of Senescent cells (SnCs) [10]. As the only cell type in cartilage, chondrocytes undergo an age-related decline in proliferation and synthetic capacity, resulting in an imbalanced metabolism of the extracellular matrix (ECM) and the progression of OA [11, 12]. Therefore, alleviating chondrocyte senescence is expected to be an effective method for treating OA.

Long non-coding RNAs (lncRNAs) are a subclass of non-coding RNAs (ncRNAs), with a length of approximately 200 nucleotides [13]. Previous studies have demonstrated the participation of lncRNAs in many human diseases, including OA [14–16]. Accumulating evidence also showed the involvement of lncRNAs in the senescence progression of multiple cells, such as fibrocytes, cardiomyocytes and vascular endothelial cells [17–19]. Hence, chondrocyte senescence may be closely related to lncRNAs, however, no research has yet focused on this area.

In the current study, we identified a novel biomarker (AC006064.4–201) in chondrocytes that was negatively correlated with the processes of chondrocyte senescence and OA development. In terms of mechanism, AC006064.4–201 could inhibit the binding of cyclin-dependent kinase inhibitor 1B (CDKN1B) mRNA to polypyrimidine tract-binding protein 1 (PTBP1), thereby downregulating CDKN1B protein translation. The AC006064.4–201/PTBP1/CDKN1B axis is expected to be a potential target for OA treatment in the future.

## Materials and methods

### Human tissue collection

This study was approved by the Ethics Committee of Sir Run Run Shaw Hospital (Hangzhou, China). Human knee joint samples were obtained from patients of different ages who had undergone total knee replacement surgery, and written informed consent was obtained from each patient. Patients with autoimmune and metabolic diseases such as hyperlipidemia, hypertension, diabetes, rheumatoid arthritis, and other diseases that affect the joints were excluded from this study. Samples were divided into two groups according to the age of the corresponding patients: samples from patients aged 50–65 years were taken as the young group, while samples from patients aged 66–80 years were taken as the old group.

### Chondrocytes isolation and culture

Human cartilage tissues were isolated from human knee joint samples and mouse cartilage tissues were isolated from mice that were 5 days old. Cartilage tissues were shredded using the physical method, washed with sterile phosphate buffered saline (PBS), and treated with 0.2% type II collagenase (Sigma-Aldrich, USA) for 24 h at 37 °C. The mixture was filtered using a 0.075 mm cell strainer and centrifuged at 1500 rpm for 10 min. Finally, the sediments were cultured evenly in Dulbecco's Modified Eagle Medium (DMEM) supplemented with 10% FBS (Thermo Fisher Scientific, Waltham, MA, USA), and maintained in an incubator set to 37 °C with 5% CO_2_ and 100% humidity.

### Animal studies

All animal experiments were approved by the Institute of Health Sciences Institutional Animal Care and Use Committee.

The adeno-associated virus (AAV) Gm49317-201 short hairpin RNA (shRNA), CDKN1B shRNA and negative control lentivirus were constructed and packaged by HanBio (Shanghai, China).

For post-traumatic arthritis animal models, forty adult male C57BL/6 mice, aged 12 weeks, were used for in vivo experiments. As a positive control, an OA model was introduced using destabilization of the medial meniscus (DMM) surgery, as previously described [20]. Briefly, 30 mice were anesthetized, and the knee joints were exposed using a medial capsular incision. The medial meniscotibial ligament (MMTL) was then transected, and the medial meniscus was displaced medially using a dissecting microscope. Finally, the joints were irrigated with sterile saline and then closed. The sham operation was performed in parallel. Briefly, 10 mice were anesthetized and the knee joint was opened, irrigated with sterile saline, and closed. One week after surgery, the unoperated mice were randomly divided into three groups (Control injection, sh Gm49317-201 injection and sh Gm49317-201 + sh CDKN1B injection) with 10 mice in each group. A total of 10 μl (approximately 1 × 10^11PFU/mL) of the control virus, Sh Gm49317-201 virus or Sh CDKN1B virus was injected into the knee joints with a microinjector. Seven weeks after the injection, the mice were sacrificed and the knee joints were separated for micro-computed tomography (micro-CT) evaluation and histological analysis.

For the natural senescence animal model, 30 adult male C57BL/6 mice, aged 4 months, were used for in vivo experiments. Briefly, the mice were randomly divided into three groups (control injection, sh Gm49317-201 injection and sh Gm49317-201 + sh CDKN1B injection) with 10 mice in each group. A total of 10 μl (approximately 1 × 10^11 PFU/mL) of the control virus, Sh Gm49317-201 virus or Sh CDKN1B virus was injected into the knee joints with a microinjector. The injection was repeated 4 months after the first injection. Four months after the second injection, the mice were sacrificed and the knee joints were separated for micro-CT evaluation and histological analysis.

### Transfection

The Antisense Oligonucleotides (ASOs) lAC006064.4–201 and Gm49317-201 were designed and constructed by RiboBio (Guangzhou, China). Lipofectamine RNAiMAX (Thermo Fisher Scientific) was used for ASO transfection, according to the manufacturer’s instructions. The sequences are listed in Supplementary Table S4.

### Virus infection

Overexpression plasmids and shRNA plasmids of PTBP1 and CDKN1B were designed and constructed by TsingkeBio (Beijing, China). Virus vectors and packaging plasmids were co-transfected into HEK-293 T cells using Lipofectamine 3000 transfection reagent (Thermo Fisher Scientific), according to the manufacturer’s instructions. The medium was changed 6 h after transfection. HEK-293 T cells were transfected for 48 h, and the medium was collected, centrifuged at 3000 rpm for 10 min, supplemented with 10 μg/mL polybrene (SolarBio), and added to human or mouse chondrocytes. Finally, the cells were selected using 2 μg/mL puromycin for 24 h.

### Western blotting analysis

Chondrocytes were lysed with radioimmunoprecipitation assay buffer (RIPA, Beyotime, China) containing 100 mM phenylmethanesulfonyl fluoride (PMSF) on ice for 20 min. Protein concentrations were determined by bicinchoninic acid (BCA) analysis (Beyotime, China). Equivalent amounts of proteins were then separated by sodium dodecyl sulphate-polycrylamide gel electrophoresis (SDS-PAGE) at different concentrations, transferred onto polyvinylidene fluoride membranes (Bio-Rad), blocked with 5% nonfat milk at room temperature for 1 h, and incubated with primary antibody at 4 °C overnight. The following day, the membranes were washed by tris-buffered saline (TBST) and incubated with a secondary antibody at room temperature for 1 h. Finally, the protein bands were visualized using FDbio-Femto ECL (Fudebio, Hangzhou, China) and a chemiluminescence system (Bio-Rad, USA). The antibodies used in this study are listed in Supplementary Table S5.

### Quantitative real-time polymerase chain reaction (qRT-PCR) analysis

Total RNA was extracted from primary chondrocytes or cartilage tissues using RNAEX reagent (Accurate Biotechnology, Hunan, China), according to the manufacturer’s instructions. Specific mRNAs were qualified using SYBR® Green Premix Pro Taq HS qPCR kit (Accurate Biotechnology, Hunan, China), according to the manufacturer’s instructions. The levels of lncRNAs and mRNAs were normalized to those of glyceraldehyde 3-phosphate dehydrogenase (GAPDH). The primers used are shown in Supplementary Table S3.

### RNA FISH

Cy3-labeled lncAC006064.4–201 and Gm49317-201 probes were designed and synthesized by HaokeBIO (Hangzhou, China). Probe signals were detected using a FISH kit (RiboBio), according to the manufacturer’s instructions. Nuclei were stained with DAPI. Images were acquired using a fluorescence microscope (Eclipse E600; Nikon Corporation, Tokyo, Japan). The Cy3-labled probes used in this study are listed in Supplementary Table S4.

### Immunofluorescence

For cell immunofluorescence (IF), chondrocytes were fixed with 4% paraformaldehyde for 30 min and permeated with 0.5% tritonX-100 for 30 min. For tissue IF, cartilage specimens were fixed in 4% paraformaldehyde for paraffin embedding and sectioned at 5 μm. Then, the cells or sections were blocked with 5% bovine serum albumin (BSA) at room temperature for 1 h and incubated with primary antibody at 4 °C overnight. After washing with PBS, cells or sections were incubated with CL594- or CL488-conjugated secondary antibodies (Proteintech Group, Rosemount, IL, USA) for 1 h. Nuclei were stained with DAPI. Images were acquired using a fluorescence microscope (Eclipse E600; Nikon Corporation, Tokyo, Japan). The fluorescence intensities were quantified as previously described [21]. The antibodies used in this study are listed in Supplementary Table S5.

### β-galactosidase

Chondrocyte senescence was determined using a Senescence β-Galactosidase Staining Kit (Beyotime Biotechnology, Shanghai, China), according to the manufacturer’s instructions. The percentage of positive cells was calculated using Image-Pro Plus 6.0 (NIH, Bethesda, MD, USA).

### RNA immunoprecipitation (RIP)

HEK-293 T cells were transfected with PTBP1 plasmid or vector. A Magna RIP RNA-Binding Protein Immunoprecipitation Kit (Millipore, Billerica, MA, USA) was used to perform RIP experiments. Briefly, approximately 1 × 10^7^ HEK-293 T cells were pelleted and resuspended in 100 μL of RIP Lysis Buffer supplemented with a protease inhibitors cocktail and ribonuclease inhibitors. Then, the cell lysates were incubated with antibody against PTBP1 (Abcam) or IgG at 4 °C overnight and treated with proteinase K buffer. Finally, the immunoprecipitated RNA were extracted using a RNeasy MinElute Cleanup Kit (Qiagen) and reverse transcribed (Accurate Biotechnology, Hunan, China). The expression levels of AC006064.4–201 were determined by qRT-PCR.

### RNA pull-down assay

Biotinylated AC006064.4–201 and Gm49317-201 probes were designed and synthesized by RiboBio (Guangzhou, China). An RNA pull-down kit (BersinBio, Guangzhou, China) were used for RNA pull-down assay. Approximately 1 × 10^7^ human or mouse chondrocytes were harvested and lysed. The AC006064.4–201, Gm49317-201 and Oligo probes were added to the magnetic beads. The cell lysates were incubated with these probe-coated beads at 4 °C overnight. The RNA–protein complexes were then eluted for western blotting analysis. The biotinylated probes used in this study are listed in Supplementary Table S4.

### Micro CT analysis

Mouse knee joint samples were fixed in 70% ethanol and scanned using a high-resolution a high-resolution micro-CT instrument (InspeXio SMX-225 CT FPD HR; Shimadzu Co. Ltd., Kyoto, Japan), according to the manufacturer’s instructions. The data were analyzed using Skyscan software.

### Histological analysis and Osteoarthritis Research Society International (OARSI) score

Cartilage specimens were fixed in 4% paraformaldehyde for paraffin embedding and sectioned at 5 μm. The sections were dehydrated and stained with Safranin-O/Fast green (Solarbio, Beijing, China), according to the manufacturer’s instructions. The Osteoarthritis Research Society International (OARSI) score was based on safranin O/fast green staining of each specimen, as previous described [22].

### Statistical analysis

Statistical analysis was performed using the SPSS version 18.0 software (IBM Corporation, USA). Data were analyzed using Student’s t-test, Fisher’s exact test, and one-way analysis of variance (ANOVA). The results are presented as the mean ± standard deviation (SD). Group differences were considered statistically different for *p* < 0.05 between groups.

## Results

### AC006064.4–201 exhibits lower expression in senescent and degenerated human chondrocytes (HCs)

**Fig. 1:**
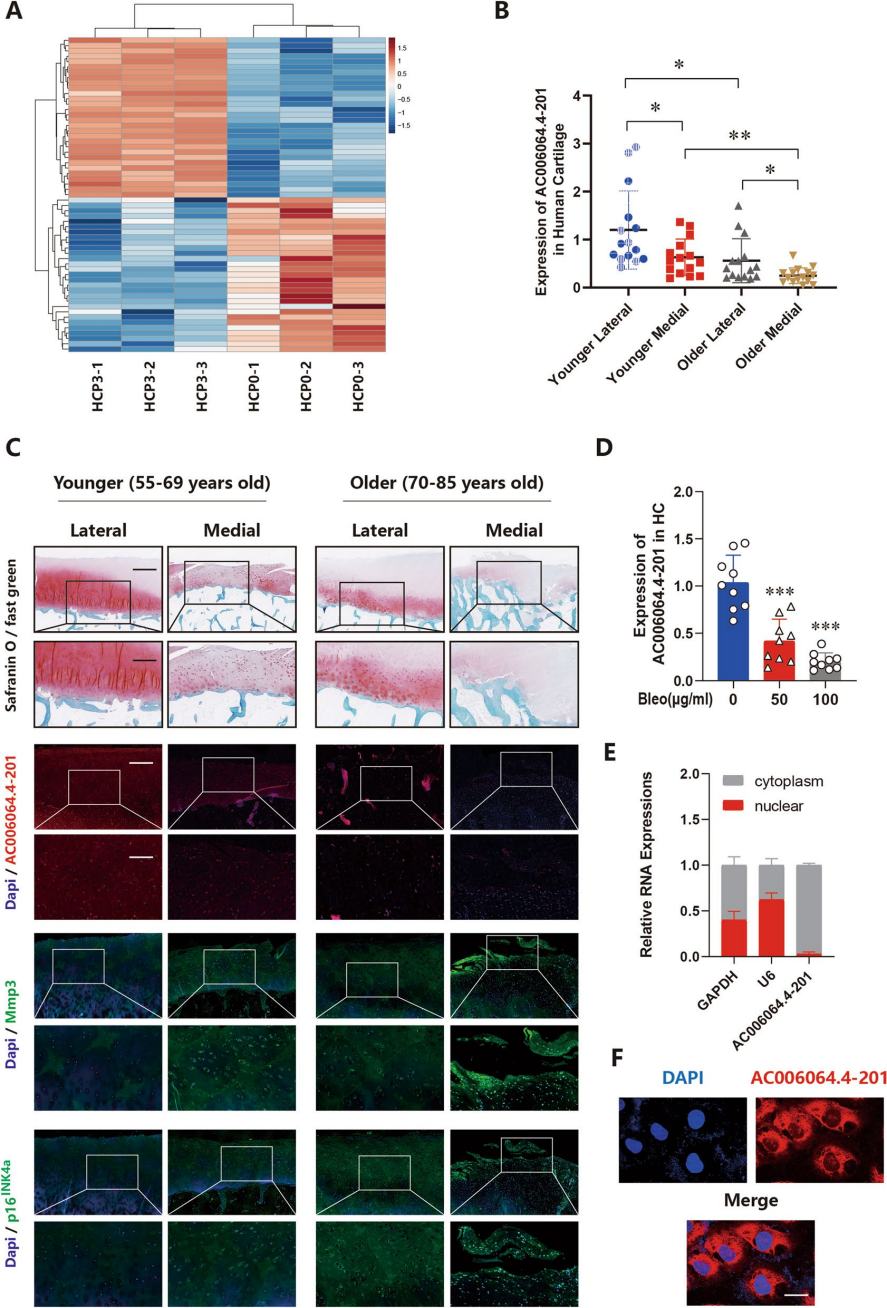
AC006064.4–201 exhibits lower expression in senescent and degenerated HCs. **A** Heat map of differentially expressed lncRNAs between normal human chondrocytes (HCP0) and senescent human chondrocytes (HCP3). **B** Quantitative real time (qRT-PCR) of AC006064.4–201 in specific sections of human knee cartilages of different ages (*n* = 15). * *p* < 0.05, ** *p* < 0.01.** C** Representative images of Safranin O / Fast green staining, FISH staining for AC006064.4–201, and IF staining for p16^INK4a^ and Mmp3 in specific sections of human knee cartilages of different ages. Scale bars, 1 mm, 500 µm and 200 µm.** D** Expression of AC006064.4–201 in HCs after cheating with different concentrations of Bleomycin (0 ug/ml, 50ug/ml and 100ug/ml) (*n* = 9, 3 donors for three replicates) *** *p* < 0.001. **E** Expression of AC006064.4–201 assessed by qRT-PCR in the nuclear and cytoplasmic fractions. **F** RNA FISH showed that AC006064.4–201 was predominantly localized in the cytoplasm. Scale bar, 25 µm

After continuous passage of human chondrocytes (HCs), we performed RNA-seq analyses on first-passaging and third-passaging chondrocytes and identified ten lncRNAs that were most differentially downregulated with the senescence of HCs (Table 1 and Fig. 1A). Among the above 10 candidates, qRT-PCR analysis showed that a novel lncRNA, AC006064.4–201, was significantly decreased in cases of both inflammation and senescence (Supplementary Fig. 1A). To further verify the expression of AC006064.4–201 in human articular cartilage, 30 human knee joint tissues were collected and divided into two groups according to age (*n* = 15), and the medial tibial plateau was compared with the lateral tibial plateau. The results demonstrated that AC006064.4–201 exhibited lower expression in the older group and decreased expression on the medial side of the tibial plateau (Fig. 1B and C, Supplementary Fig. 1B). Safranin O/Fast green staining showed more severe wear on the medial side of the tibial plateau in each group (Fig. 1C). According to the results of immunofluorescence (IF) staining, compared to the younger group and the lateral cartilage, both Mmp3 and p16^INK4a^ were higher in the older group and the medial cartilage (Fig. 1C, Supplementary Fig. 1B). Different concentrations of bleomycin and doxorubicin were used to induce human chondrocyte senescence in vitro, and the results indicated that AC006064.4–201 exhibited a concentration-related downward trend (Fig. 1D, Supplementary 1C). Finally, nuclear separation experiments coupled with qRT-PCR analysis and RNA FISH revealed that AC006064.4–201 was predominantly localized to the cytoplasm (Fig. 1E and F). Cumulatively, these results demonstrated AC006064.4–201 was downregulated in senescent and degenerated HCs and thus may play an important role in chondrocyte senescence and OA progression.

### AC006064.4–201 alleviates the senescence of HCs

**Fig. 2:**
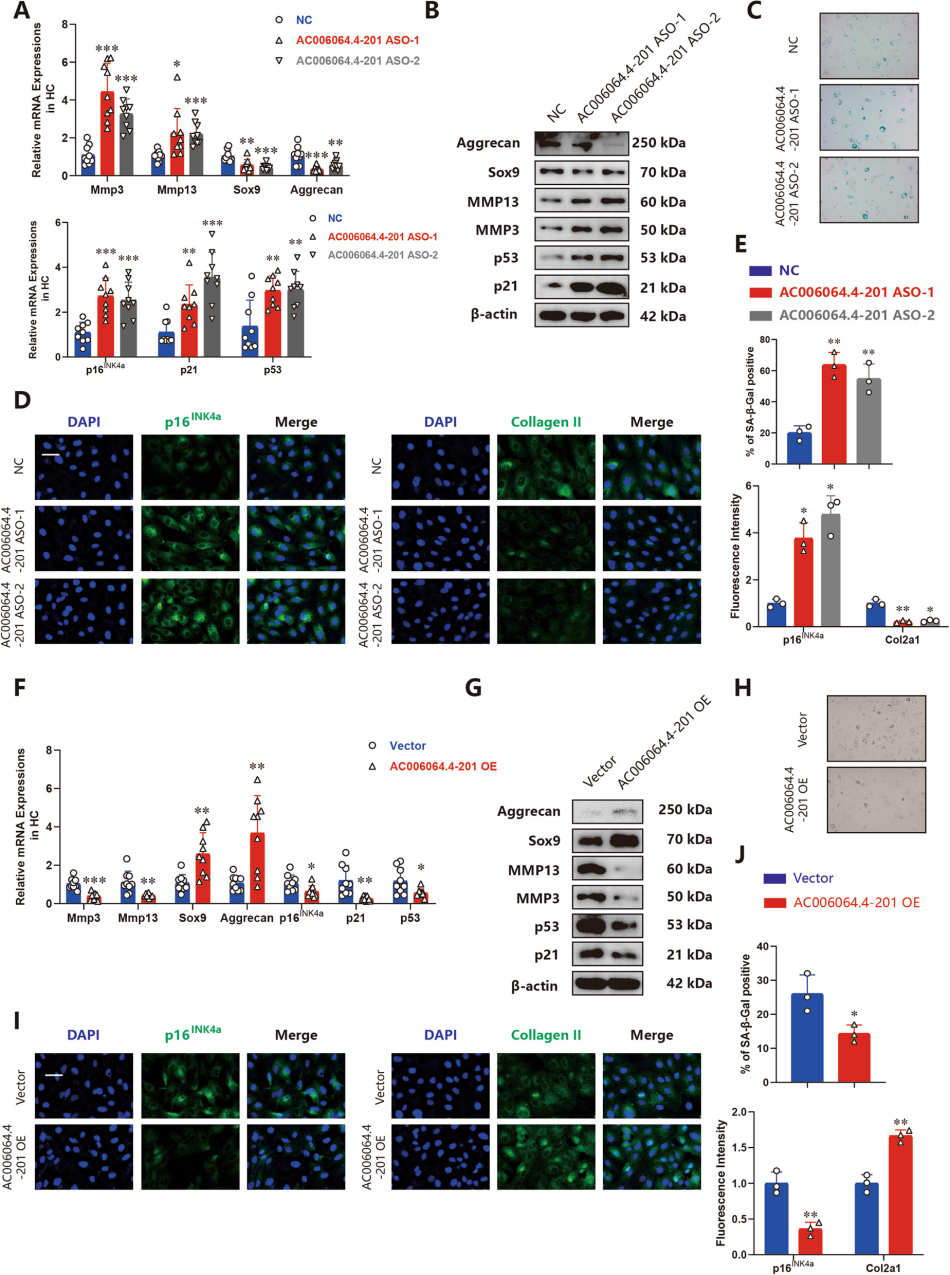
AC006064.4–201 alleviates the senescence of HCs. **A** MRNA levels of Mmp3, Mmp13, Sox9, Aggrecan, p16^INK4a^, p21 and p53 assessed by qRT-PCR in HCs after treating with AC006064.4–201 Antisense Oligonucleotides (ASOs) (*n* = 9, 3 donors for three replicates) * *p* < 0.05, ** *p* < 0.01, *** *p* < 0.001. **B** Protein levels of Mmp3, Mmp13, Sox9, Aggrecan, p21 and p53 assessed by western blotting in HCs after treating with AC006064.4–201 ASOs. **C** Representative images of β-galactosidase staining in HCs after treating with AC006064.4–201 ASOs. **D** Representative photomicrographs of IF staining of p16^INK4a^ and Col2a1 when AC006064.4–201 was downregulated in HCs. Scale bar, 50 µm.** E** Quantification of SA-β-Gal staining and fluorescence intensity of IF staining of p16^INK4a^ and Col2a1 in the above three groups. * *p* < 0.05, ** *p* < 0.01.** F** MRNA levels of Mmp3, Mmp13, Sox9, Aggrecan, p16^INK4a^, p21 and p53 assessed by qRT-PCR when AC006064.4–201 was upregulated in HCs (*n* = 9, 3 donors for three replicates) * *p* < 0.05, ** *p* < 0.01, *** *p* < 0.001. **G** Protein levels of Mmp3, Mmp13, Sox9, Aggrecan, p21 and p53 assessed by western blotting when AC006064.4–201 was overexpressed in HCs. **H** Representative images of β-galactosidase staining in HCs when AC006064.4–201 was overexpressed in HCs. **I** Representative photomicrographs of IF staining of p16^INK4a^ and Col2a1 when AC006064.4–201 was upregulated in HCs. Scale bar, 50 µm.** J** Quantification of SA-β-Gal staining and fluorescence intensity of IF staining of p16^INK4a^ and Col2a1 in the above two groups. * *p* < 0.05, ** *p* < 0.01

To further investigate whether AC006064.4–201 is involved in the regulation of HCs senescence, two AC006064.4–201 Antisense Oligonucleotides (ASOs) that could specifically and stably knock down AC006064.4–201 in HCs were used (Supplementary Fig. 2A). qRT-PCR analysis demonstrated that knockdown of AC006064.4–201 in HCs resulted in significantly increased mRNA levels of cell senescence factors (p16^INK4a^, p21, and p53) and degradation enzymes (Mmp3 and Mmp13) and reduced mRNA levels of Sox9, Aggrecan and Col2a1 (Fig. 2A). The results of western blotting and IF staining were consistent with the results of qRT-PCR analysis (Fig. 2B, D and E). Moreover, β-galactosidase staining analysis was performed to detect senescent HCs, and the results indicated that the knockdown of AC006064.4–201 considerably enhanced the number of senescent HCs (Fig. 2C and E).

Subsequently, we studied the therapeutic effect of AC006064.4–201 in HCs. An overexpression plasmid was constructed and transfected to upregulate AC006064.4–201 expression (Supplementary Fig. 2B). qRT-PCR analysis, western blotting and IF staining showed that the increase of AC006064.4–201 reduced the levels of p16^INK4a^, p21, p53, Mmp3 and Mmp13, however, it promoted the expression of Sox9, Aggrecan and Col2a1 (Fig. 2F, G, I and J). Additionally, the number of senescent HCs decreased with the upregulation of AC006064.4–201 (Fig. 2H and J).

Taken together, these results illustrated that AC006064.4–201 could protect against OA by alleviating the senescence and degeneration of HCs.

### AC006064.4–201 directly interacts with PTBP1 in HCs

**Fig. 3:**
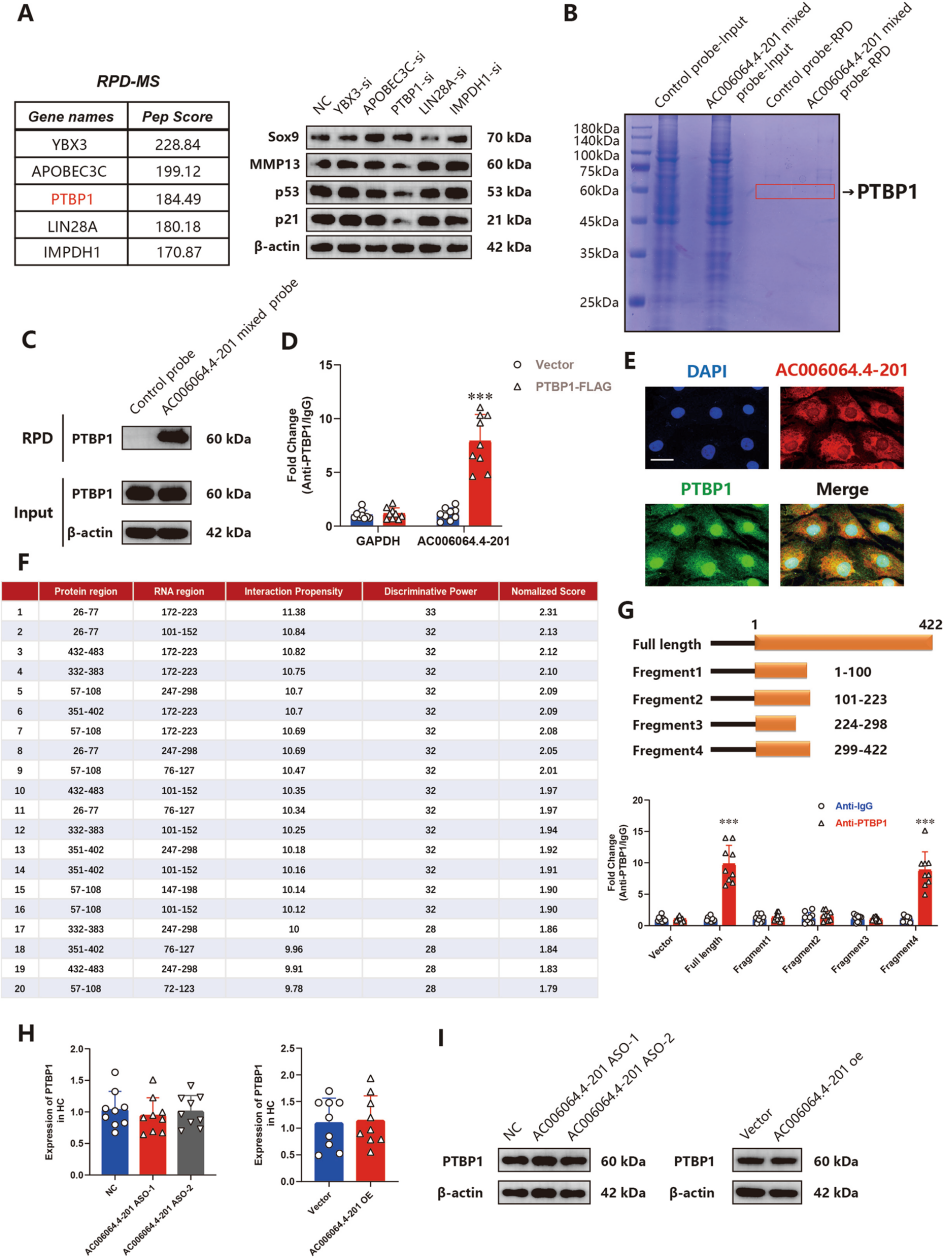
AC006064.4–201 directly interacts with Polypyrimidine tract-binding protein 1 (PTBP1) in HCs. **A** Five proteins with highest pep score of AC006064.4–201 RPD-MS and their effects on Mmp13, Aggrecan, p21 and p53 assessed by western blotting analysis. **B** Coomassie brilliant blue staining of proteins binding to AC006064.4–201. **C** Western blotting of PTPB1 in HCs after pulled-down with the biotinylated AC006064.4–201 mixed probes. **D** RNA immunoprecipitation (RIP) assay for AC006064.4–201 levels in HEK-293 T cells transfected with PTBP1-Flag (*n* = 9, 3 donors for three replicates) *** *p* < 0.001. **E** RNA–protein colocalization assay confirmed the interaction between AC006064.4–201 and PTBP1 in HCs. Scale bar, 25 µm. **F** Interacting regions between AC006064.4–201 and PTBP1 predicted by catRAPID tool. **G** Schematic diagram of truncated AC006064.4–201, and RIP assay identified the binding sequence of AC006064.4–201 for PTBP1 (*n* = 9, 3 donors for three replicates) *** *p* < 0.001. **H** MRNA levels of PTBP1 assessed by qRT-PCR when AC006064.4–201 was knocked down or upregulated in HCs (*n* = 9, 3 donors for three replicates) **I** Protein levels of PTBP1 assessed by western blotting analysis when AC006064.4–201 was knocked down or upregulated in HCs

Studies have revealed that lncRNAs are associated with a plethora of cellular functions, but most of them require interactions with one or more RNA-binding proteins (RBPs) [23–26]. To identify the proteins that interacted with AC006064.4–201, three biotinylated AC006064.4–201 probes at different sites were synthesized and mixed together. Then RPD-MS was employed, and 100 proteins were identified (Table 2, Fig. 3B). Five of the highest pep- score proteins were selected and knocked down to verify their functions (Supplementary Fig. 2C). The results revealed that only PTBP1 affected the expression of p21, p53, Mmp13 and Sox9 (Fig. 3A). Therefore, PTBP1 was selected for further analyses. The binding of AC006064.4–201 to PTBP1 was confirmed by RNA pull-down and RIP assay (Fig. 3C and D). The interaction between AC006064.4–201 and PTBP1 was verified with RNA–protein colocalization in HCs (Fig. 3E). To further study the binding sites of AC006064.4–201 and PTBP1, we used the catRAPID tool to predict the interacting regions (Fig. 3F) and truncated FL AC006064.4–201 into four segments (S1: 1–100 nt, S2: 101–223 nt, S3: 224–298 nt, S4: 299–422 nt) according to the predicted binding sites. The results of the RIP assay indicated that only FL and S4 were pulled down by PTBP1 (Fig. 3G). Taken together, these results demonstrated that AC006064.4–201 directly interacts with PTBP1 in HCs.

Subsequently, we explored whether AC006064.4–201 affects PTBP1 expression. However, qRT-PCR analysis and western blotting showed that both the mRNA and protein levels of PTBP1 did not change when AC006064.4–201 was knocked down or overexpressed in the HCs (Fig. 3H and I). Therefore, we hypothesized that AC006064.4–201 functions by influencing the downstream target of PTBP1 and conducted follow-up research.

### AC006064.4–201 blocks PTBP1 from binding to CDKN1B mRNA

**Fig. 4:**
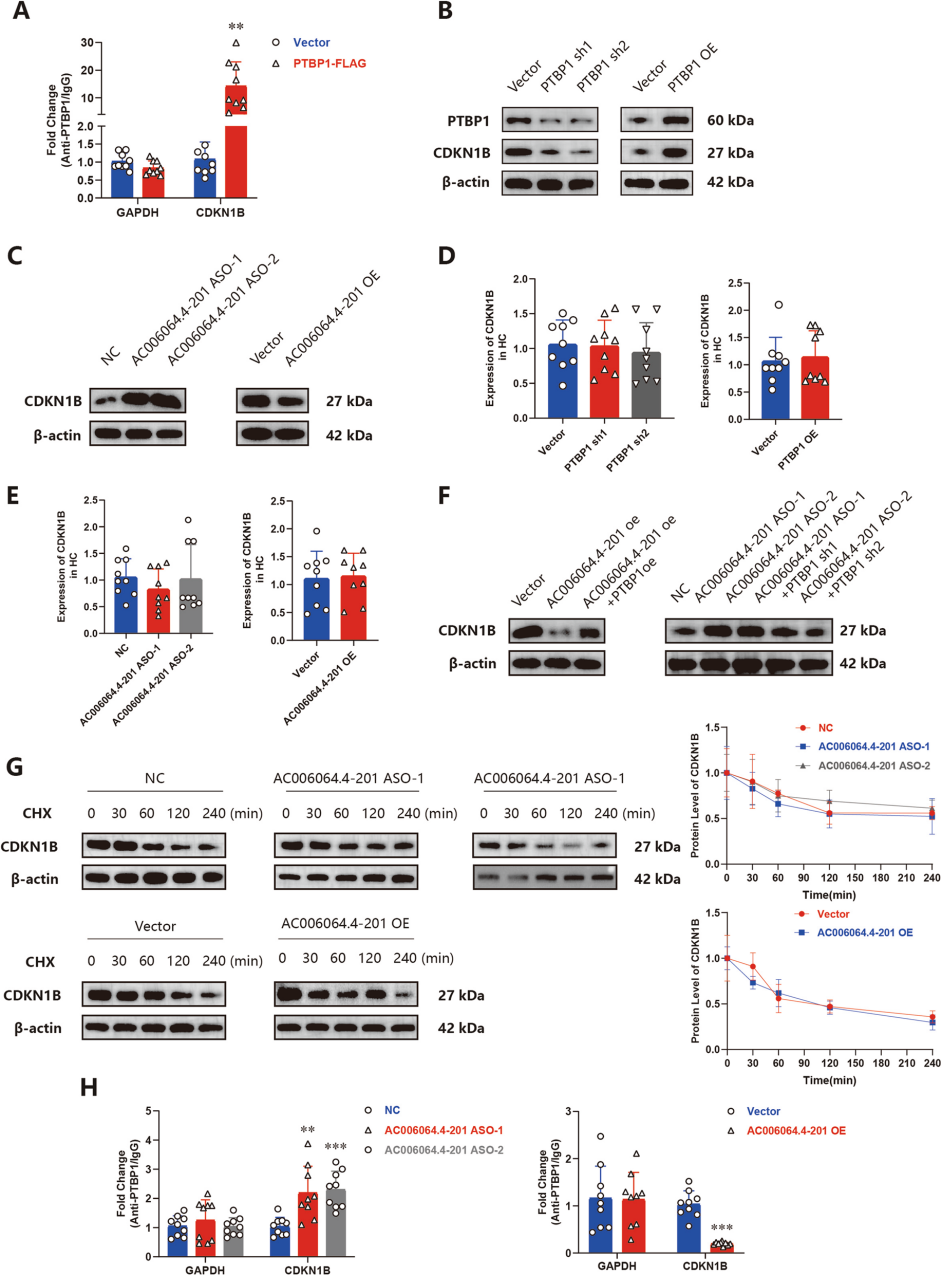
AC006064.4–201 blocks PTBP1 from binding to CDKN1B mRNA. **A** RIP assay for PTPB1 levels in HEK-293 T cells transfected with PTBP1-Flag (*n* = 9, 3 donors for three replicates) ** *p* < 0.01. **B** Protein levels of CDKN1B assessed by western blotting analysis when PTBP1 was knocked down or upregulated in HCs. **C** Protein levels of CDKN1B assessed by western blotting analysis when AC006064.4–201 was knocked down or upregulated in HCs. **D** MRNA levels of CDKN1B assessed by qRT-PCR when PTBP1 was knocked down or upregulated in HCs (*n* = 9, 3 donors for three replicates) **E** MRNA levels of CDKN1B assessed by qRT-PCR when AC006064.4–201 was knocked down or upregulated in HCs (*n* = 9, 3 donors for three replicates) **F** Western blotting of CDKN1B when AC006064.4–201 and PTBP1 were simultaneously knocked down or upregulated in HCs. **G** AC006064.4–201 was upregulated or knocked down in HCs first, and treated with 50 mg/mL CHX for the indicated times, finally the protein levels of CDKN1B were assessed by western blotting analysis. **H** RIP assay revealed the combination levels between CDKN1B and PTBP1 when AC006064.4–201 was upregulated or knocked down in HEK-293 T cells (*n* = 9, 3 donors for three replicates) ** *p* < 0.01, *** *p* < 0.001

PTBP1 was shown to bind to the mRNA of CDKN1B and improve its stability [27]. This was also verified in our study by the RIP assay (Fig. 4A). We subsequently tested whether CDKN1B is the downstream target of the AC006064.4–201/PTBP1 axis. The results of western blotting indicated that the protein level of CDKN1B was increased when PTBP1 was overexpressed or AC006064.4–201 was knocked down, which was similar to the opposite trend (Fig. 4B and C). qRT-PCR analysis revealed that AC006064.4–201 and PTBP1 had no effect on the mRNA level of CDKN1B (Fig. 4D and E). Furthermore, when AC006064.4–201 and PTBP1 were both downregulated or both overexpressed, the effect of AC006064.4–201 on CDKN1B was reversed (Fig. 4F). These results suggest that AC006064.4–201 influences the protein synthesis of CDKN1B by improving the stability of CDKN1B mRNA. To confirm this hypothesis, cycloheximide (CHX) was used to block protein synthesis. The results confirmed that, when the protein synthesis process was blocked, the effects of AC006064.4–201 on CDKN1B disappeared (Fig. 4G). Finally, the RIP assay indicated that the interaction between PTBP1 and CDKN1B was enhanced when AC006064.4–201 was knocked down and decreased when AC006064.4–201 was upregulated (Fig. 4H). Together, these results demonstrated that AC006064.4–201 blocks PTBP1 from binding to CDKN1B mRNA and reduces the protein synthesis of CDKN1B by reducing its mRNA stability.

### CDKN1B mediates the AC006064.4–201/PTBP1 axis in HCs

**Fig. 5:**
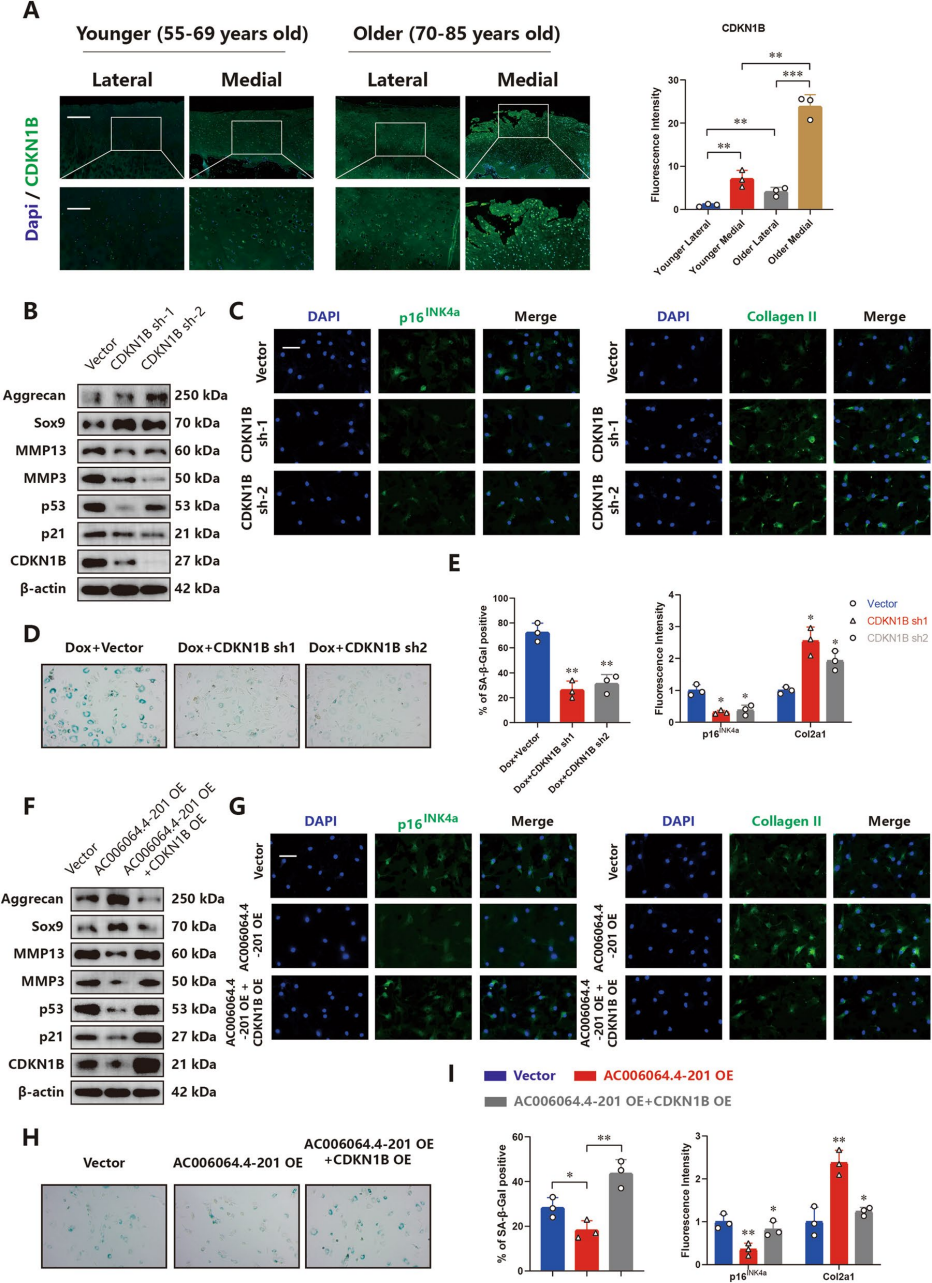
CDKN1B mediates the AC006064.4–201 / PTBP1 axis in HCs.** A** Representative images and fluorescence intensity of IF staining for CDKN1B in specific sections of human knee cartilages of different ages. ** *p* < 0.01, *** *p* < 0.001. Scale bars, 500 µm and 200 µm. **B** Protein levels of Mmp3, Mmp13, Sox9, Aggrecan, CDKN1B, p21 and p53 assessed by western blotting when CDKN1B was knocked down in HCs.** C** Representative images of IF staining for p16^INK4a^ and Col2a1 when CDKN1B was downregulated in HCs. Scale bar, 50 µm. **D** Representative images of β-galactosidase staining in HCs when CDKN1B was downregulated. **E** Quantification of SA-β-Gal staining and fluorescence intensity of IF staining of p16^INK4a^ and Col2a1 in the above two groups. * *p* < 0.05, ** *p* < 0.01. **F** Protein levels of Mmp3, Mmp13, Sox9, Aggrecan, CDKN1B, p21 and p53 assessed by western blotting when AC006064.4–201 and CDKN1B were co-overexpressed in HCs. **G** Representative images of IF staining for p16^INK4a^ and Col2a1 when AC006064.4–201 and CDKN1B were co-overexpressed in HCs. Scale bar, 50 µm. **H** Representative images of β-galactosidase staining in HCs when AC006064.4–201 and CDKN1B were co-overexpressed.** I** Quantification of SA-β-Gal staining and fluorescence intensity of IF staining of p16.^INK4a^ and Col2a1 in the above two groups. * *p* < 0.05, ** *p* < 0.01

We examined CDKN1B levels in human cartilage tissues, and the IF staining results indicated that CDKN1B expression was higher in the older group and increased on the medial side of the tibial plateau (Fig. 5A). To further investigate the role of CDKN1B in OA, two shRNAs targeting CDKN1B were designed, and their knockout efficiency was verified (Supplementary Fig. 2D). qRT-PCR, western blotting and IF staining showed that knockdown of CDKN1B resulted in a decrease in p16^INK4a^, p21, p53, Mmp3 and Mmp13, while expression of Sox9, Aggrecan and Col2a1 was increased (Fig. 5B, C and E, Supplementary Fig. 2E). The number of senescent HCs declined with CDKN1B downregulation (Fig. 5D and E). These results demonstrated that CDKN1B contributes to the senescence and degeneration of HCs. AC006064.4–201 and CDKN1B were simultaneously overexpressed to assess whether CDKN1B could antagonize the function of AC006064.4–201 in HCs. qRT-PCR, western blotting and IF staining indicated that upregulated CDKN1B could reverse the altered levels of p16^INK4a^, p21, p53, Mmp3, Mmp13, Sox9, Aggrecan and Col2a1 caused by overexpression of AC006064.4–201 (Fig. 5F, G and I, Supplementary Fig. 2F and G). Additionally, the reduced senescent HCs was also reversed, as shown by β-galactosidase staining analysis (Fig. 5H and I).

Taken together, these results revealed that CDKN1B mediates the AC006064.4–201/PTBP1 axis and contributes to the senescence and degeneration of HCs.

### AC006064.4–201 (Gm49317-201)/PTBP1/CDKN1B axis is conserved between humans and mice

**Fig. 6:**
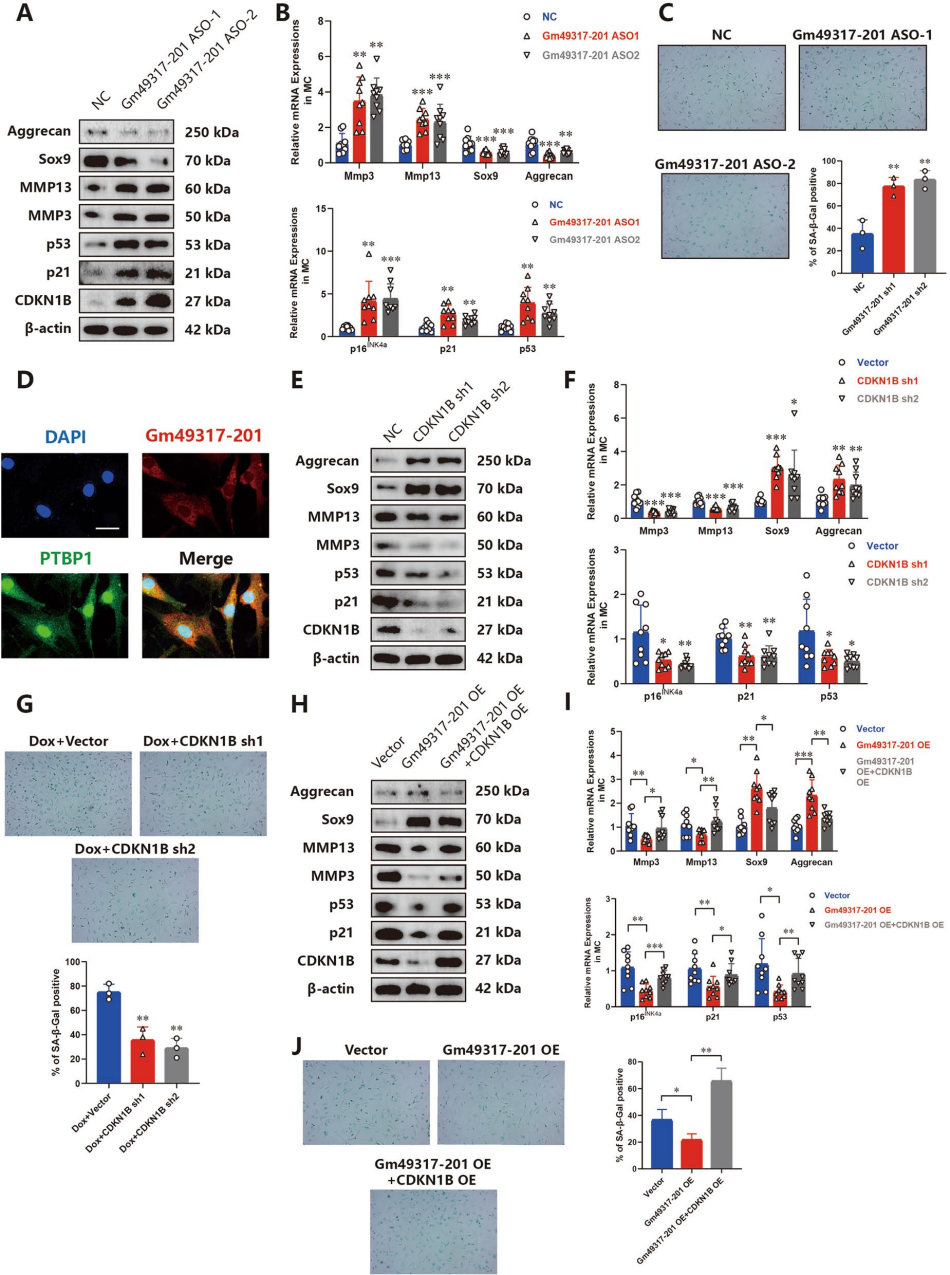
AC006064.4–201 (Gm49317-201) / PTBP1 / CDKN1B axis was conserved between human and mice. **A** Western blotting analysis of Mmp3, Mmp13, Sox9, Aggrecan, CDKN1B, p21 and p53 in MCs after treating with Gm49317-201 ASOs.** B** MRNA levels of Mmp3, Mmp13, Sox9, Aggrecan, CDKN1B, p21 and p53 assessed by qRT-PCR in MCs after treating with Gm49319 ASOs (*n* = 9, 3 donors for three replicates) ** *p* < 0.01, *** *p* < 0.001. **C** Representative images and quantification of β-galactosidase staining in the above three groups. ** *p* < 0.01. **D** RNA–protein colocalization assay confirmed the interaction between Gm49317-201 and PTBP1 in MCs. Scale bar, 25 µm.** E** Western blotting analysis of Mmp3, Mmp13, Sox9, Aggrecan, CDKN1B, p21 and p53 in MCs after knocking down of CDKN1B. **F** MRNA levels of Mmp3, Mmp13, Sox9, Aggrecan, CDKN1B, p21 and p53 assessed by qRT-PCR in MCs after knocking down of CDKN1B (*n* = 9, 3 donors for three replicates) * *p* < 0.05, ** *p* < 0.01, *** *p* < 0.001. **G** Representative images and quantification of β-galactosidase staining of MCs after knocking down of CDKN1B. ** *p* < 0.01.** H** Western blotting analysis of Mmp3, Mmp13, Sox9, Aggrecan, CDKN1B, p21 and p53 in MCs after co-upregulation of Gm49317-201 and CDKN1B. **I** MRNA levels of Mmp3, Mmp13, Sox9, Aggrecan, CDKN1B, p21 and p53 assessed by qRT-PCR in MCs after co-upregulation of Gm49317-201 and CDKN1B (*n* = 9, 3 donors for three replicates) * *p* < 0.05, ** *p* < 0.01, *** *p* < 0.001. **J** Representative images and quantification of β-galactosidase staining in the above three groups. * *p* < 0.05, ** *p* < 0.01

To determine the need for in vivo experiments in mice, we studied whether there is a conserved lncRNA in mice that could regulate the PTBP1/CDKN1B axis. According to the Ensemble Database, a similar lncRNA, named Gm49317-201, exists in mice and has 179 bases identical to AC006064.4–201 (Supplementary Fig. 3A). Interestingly, the binding sites of AC006064.4–201 and PTBP1 are located in the same base sequences. Therefore, we speculated that Gm49317-201 has the same function as AC006064.4–201 in chondrocytes. Two Gm49317-201 ASOs were generated that specifically and stably knock down Gm49317-201 in mouse chondrocytes (MCs) (Supplementary Fig. 3B). Western blotting and qRT-PCR analysis showed that the knockdown of Gm49317-201 resulted in an increase of p16^INK4a^, p21, p53, Mmp3 and Mmp13, while the expressions of Sox9, Aggrecan and Col2a1 were decreased, the protein level of CDKN1B was increased, and the mRNA was unchanged (Fig. 6A and B). The number of senescent MCs increased when Gm49317-201 was downregulated (Fig. 6C). These results indicate that Gm49317-201 can alleviate the senescence and degeneration of MCs.

We then confirmed the binding of Gm49317-201 to PTBP1 using an RNA pull-down assay (Supplementary Fig. 3C). The interaction between Gm49317-201 and PTBP1 was also verified by RNA–protein colocalization in MCs (Fig. 6D). Subsequently, two shRNAs targeting CDKN1B in mice were designed, and their knockout efficiency was verified (Supplementary Fig. 3D). The role of CDKN1B in MCs senescence was tested by western blotting, qRT-PCR analysis and β-galactosidase staining, which was similar to that in HCs (Fig. 6E, F and G). Finally, Gm49317-201 and CDKN1B were both overexpressed in MCs, demonstrating that the overexpression of CDKN1B could antagonize the function of Gm4937-201 in MCs (Fig. 6H, I and J).

In summary, these results revealed that the AC006064.4–201 (Gm49317-201) / PTBP1 / CDKN1B axis is conserved between humans and mice. Moreover, AC006064.4–201 and Gm49317-201 had similar functions in HCs and MCs. This prompted us to conduct further in vivo experiments using mice.

### Gm49317-201 and CDKN1B affect both age-related and post-traumatic OA in vivo

**Fig. 7:**
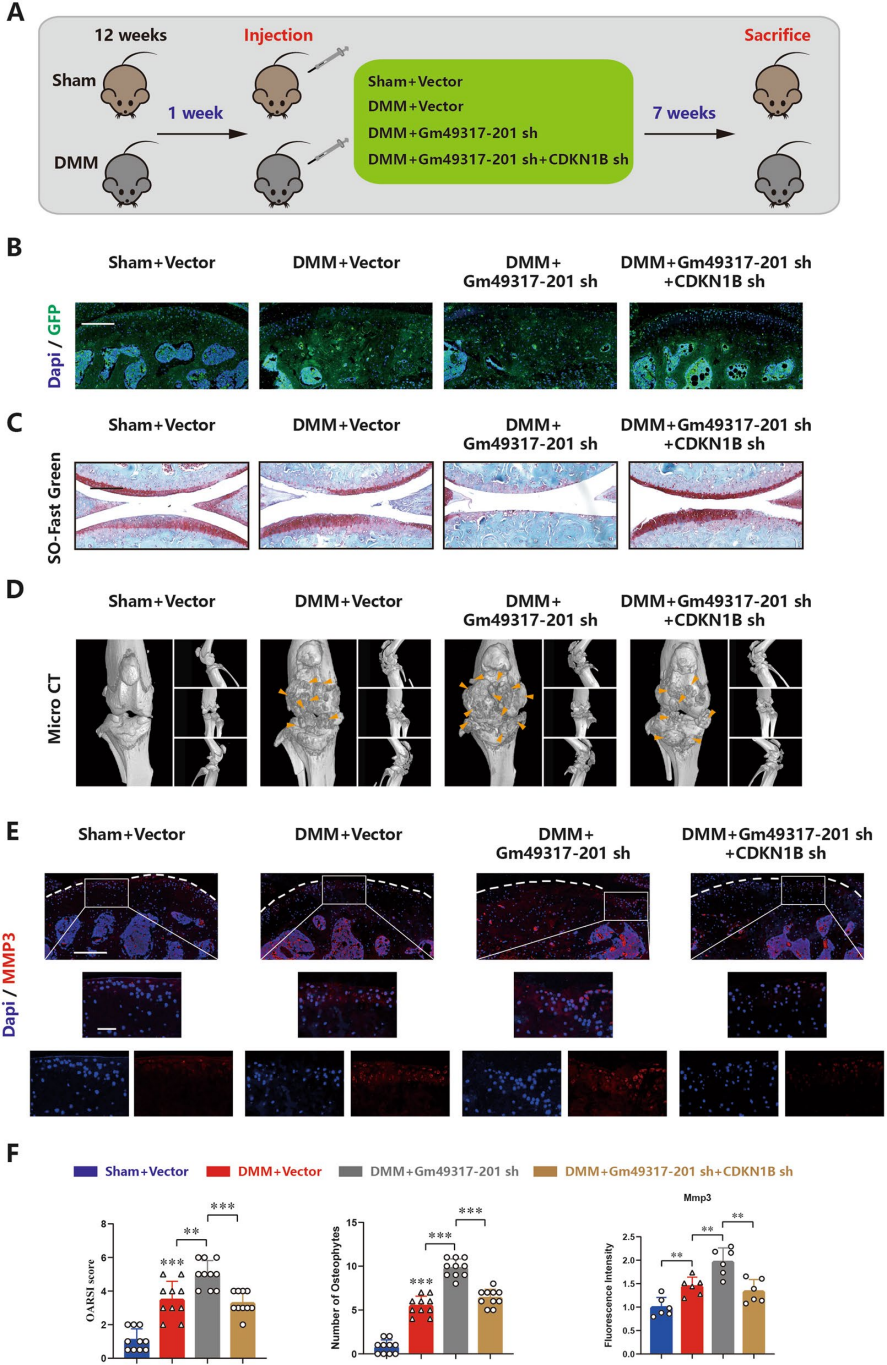
Gm49317-201 and CDKN1B affects post-traumatic OA in vivo. **A** Establishment of a rescue model for mice post-traumatic OA targeting Gm49317-201 and CDKN1B. **B** Representative images of GFP staining revealed the specific adeno-associated virus (AAV) efficiently infected the cartilage of different groups. Scale bar, 200 µm. **C** Representative images of Safranin O / Fast green staining in mice cartilage of different groups. Scale bar, 200 µm. **D** 3D reconstruction images of micro-CT scanning of the knees and osteophytes (yellow arrow). **E** Representative images of IF staining for Mmp3 in mice cartilage of different groups. Scale bars, 200 µm and 50 µm.** F** Fluorescence intensity of FISH staining and IF staining, OARSI grade according to Safranin O / Fast green staining and number of osteophytes for micro-CT scanning in mice cartilage of different groups (*n* = 10) ** *p* < 0.01, *** *p* < 0.001

To investigate the role of Gm49317-201 in post-traumatic OA, a mouse model was introduced in this study, as described in the Methods section (Fig. 7A). The mice were divided into four groups, and the specific AAV efficiently infected the cartilage in these groups (Fig. 7B). Safranin O/fast green staining showed that the cartilage layer was thinner in DMM mice than in sham-operated mice, and the injection of Gm49317-201 shRNA aav aggravated the damage to the cartilage layer caused by DMM surgery, this deterioration was rescued by the injection of CDKN1B shRNA aav (Fig. 7C and F). 3D reconstruction of the micro-CT of mouse knees revealed more osteophytes in the DMM + Vector and DMM + Gm49317-201 sh groups than in the sham and DMM + Gm49317-201 sh + CDKN1B sh groups (Fig. 7D and F). The expression of CDKN1B, Mmp3 and Col2a1 were determined by IF staining, and the results indicated that DMM + Vector and DMM + Gm49317-201 sh groups exhibited higher expression of CDKN1B and Mmp3 than the sham and DMM + Gm49317-201 sh + CDKN1B sh groups, whereas the expression of Col2a1 was the opposite (Fig. 7E and F, Supplementary Fig. 4A). These results demonstrated that decreasing the expression of Gm49317-201 in mouse cartilage aggravates post-traumatic OA, and this effect was mediated by upregulation of CDKN1B protein levels.

**Fig. 8:**
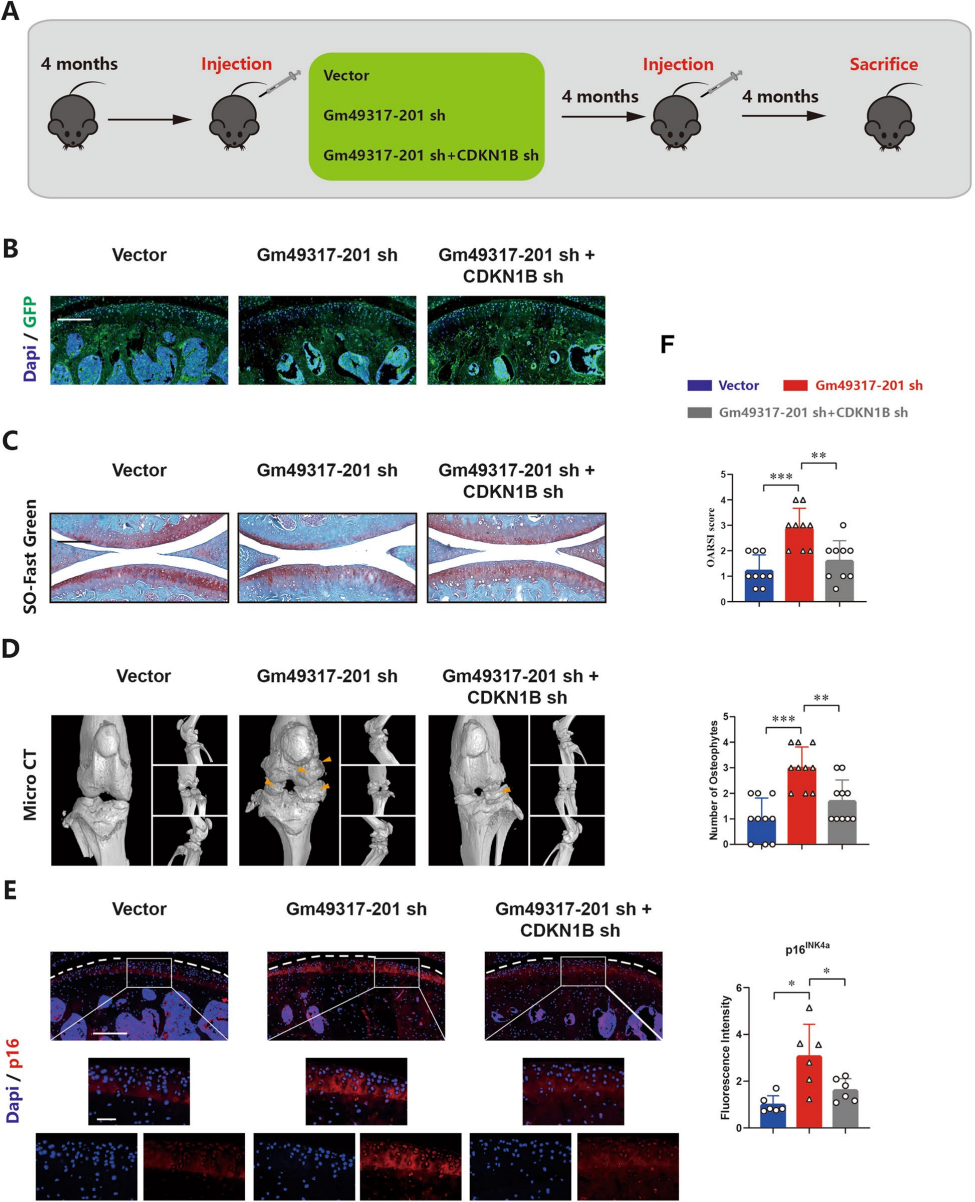
Gm49317-201 and CDKN1B affects age-related OA in vivo. **A** Establishment of a rescue model for mice age-related OA targeting Gm49317-201 and CDKN1B. **B** Representative images of GFP staining revealed the specific adeno-associated virus (AAV) efficiently infected the cartilage of different groups. Scale bar, 200 µm. **C** Representative images of Safranin O / Fast green staining and OARSI grade use for evaluation of cartilage degeneration in mice cartilage of different groups (*n* = 10) ** *p* < 0.01, *** *p* < 0.001. Scale bar, 200 µm.** D** 3D reconstruction images of micro-CT scanning of the knees and the number of osteophytes (yellow arrow) (*n* = 10) ** *p* < 0.01, *** *p* < 0.001. **E** Representative images and Fluorescence intensity of IF staining for p16INK4a in mice cartilage of different groups (*n* = 10) * *p* < 0.05. Scale bars, 200 µm and 50 µm

To investigate the role of Gm49317-201 in age-related OA, another mouse model was used in this study, as described in the Methods section (Fig. 8A). We divided the mice into three groups and proved the efficient infection of specific AAV on the cartilage in these groups (Fig. 8B). Safranin O/fast green staining showed that the cartilage layer was thinner in the Gm49317-201 sh group than in the Vector and Gm49317-201 sh + CDKN1B sh groups (Fig. 8C). More osteophytes appeared in the Gm49317-201 sh group than in the other two groups (Fig. 8D). In addition, IF staining revealed that the Gm49317-201 sh group exhibited higher expression of CDKN1B and p16^INK4a^ and lower expression of Col2a1 than the other two groups (Fig. 8E and Supplementary Fig. 5A). These results demonstrated that decreasing the expression of Gm49317-201 in mouse cartilage would accelerate the senescence of mouse cartilage and lead to the earlier occurrence of OA in mice.

## Discussion

Currently, OA is an incurable orthopedic disease. Current treatment strategies for OA, including pharmacological treatments, regenerative treatments and joint replacement surgery, are limited to relieving symptoms rather than slowing the biological process of OA progression [28, 29]. Therefore, new treatment strategies are needed, and the field would benefit from a deeper understanding of the mechanisms underlying OA development and progression.

As an age-related or post-traumatic disease, OA is primarily caused by an imbalance in matrix anabolism and catabolism in cartilage [30–32]. Recent studies have considered chondrocytes senescence as an important cellular event contributing to matrix catabolism, leading to the degradation of the cartilage matrix and OA development [8, 33]. Which was also confirmed by our present study, as we found the expression of p16^INK4a^ and Mmp3 were increased in cartilage tissues of elderly patients and those with severe wear, it proves the closely relationship between chondrocyte senescence and OA progression. Furthermore, in vivo experiments in mice conducted by previous researches revealed that the local clearance of senescent cells in mouse cartilage attenuated the development of post-traumatic OA and created a pro-regenerative environment [34, 35]. Therefore, relieving chondrocyte senescence is expected to become a new strategy for OA treatment in the future.

LncRNAs are often found in mammalian epigenetic systems and have been shown to participate in various cellular events, including senescence, inflammation, proliferation, metastasis and apoptosis [23, 36–38]. Recent studies have indicated that lncRNAs involved in OA development [13, 15, 39]. We identified that AC006064.4–201 was closely related to chondrocyte senescence and OA development. AC006064.4–201 is a novel lncRNA that has not been reported before. Herein, we found that AC006064.4–201 is downregulated in senescent and degenerated human cartilage. Moreover, bleomycin and doxorubicin were used to induce HCs senescence as previously reported [40], and both of them could decrease the expression of AC006064.4–201 in HCs. In vitro functional experiments indicated that AC006064.4–201 alleviates the senescence and degeneration in HCs. For in vivo experiments, two mouse models were introduced to separately imitate post-traumatic OA and age-related OA. Knocking down the corresponding lncRNA Gm49317-201 in mouse cartilage resulted in more severe cartilage damage in DMM mice and a much earlier occurrence of OA in normal-growing mice. Therefore, AC006064.4–201 could be used as a novel biomarker of cartilage senescence and degeneration, and proved to be a protective factor against the OA development.

Complex formation within proteins has been proved to be an important mechanism for lncRNAs to perform biological functions [24, 41]. We subsequently explored the proteins that could interact with AC006064.4–201 through RPD-MS and identified PTBP1.

As a member of the heterogeneous nuclear ribonucleoproteins (hnRNPs) family, PTBP1 is a widely studied RNA binding protein that binds to the polypyrimidine sequence on the pre-mRNA, and involves in regulating mRNA splicing, translation, stability and localization [42, 43]. Previous studies have indicated that PTBP1 plays an important role in cancer progression [44, 45], Alzheimer’s disease [46, 47] and cardiac fibrosis [48]. However, until now, there has been no study on PTBP1 in chondrocyte senescence and OA progression. Our research found that AC006064.4–201 could directly interact with PTBP1 in HCs but had no effect on the expression of PTBP1, including the mRNA and protein levels. We therefore explored if AC006064.4–201 functions by influencing the downstream target of PTBP1. And the results further revealed that PTBP1 was able to bind to the mRNA of CDKN1B and improve its stability, resulting in the increased translation of CDKN1B protein. Mounting evidence suggested that CDKN1B is a key regulator of cell cycle progression, which was recognized as an important senescence marker in aging-related diseases such as osteoporosis [49, 50], atherosclerosis [51] and Alzheimer’s disease [52]. In the present study, we performed functional experiments and reported that CDKN1B is positively correlated with chondrocyte senescence and OA progression. Besides, AC006064.4–201 was shown to reduce the expression of CDKN1B by blocking the binding between PTBP1 and CDKN1B mRNA. And co-overexpression experiments indicated that the function of AC006064.4–201 could be antagonized by CDKN1B in HCs. Hence, CDKN1B serves as an important downstream target of the AC006064.4–201/PTBP1 axis. In vivo experiments revealed that the downregulation of CDKN1B in mouse cartilage could alleviate OA in DMM mice and delay cartilage senescence in normal-growing mice.

Taken together, we evaluated the function of the AC006064.4–201/PTBP1/CDKN1B axis in cartilage senescence and degeneration in this study. However, aberrant subchondral remodeling and synovitis are also crucial aspects of OA progression, which will be explored in future studies.

## Conclusions

In summary, our research revealed a novel molecular marker, AC006064.4–201, that was found to protect against OA by alleviating senescence and degeneration of cartilage. Mechanistically, AC006064.4–201 could destabilize CDKN1B mRNA by interacting with PTBP1 and decreasing the protein expression of CDKN1B. Cumulatively, this study provides new molecular targets for the early diagnosis and treatment of OA.

## Supplementary Information

**Additional file 1:** **Supplementary Figure 1. (A) **Expressionof ten lncRNAs in HCs assessed by qRT-PCR after treating with 10ng/ml IL-1β. (*n*=9, 3 donors for three replicates) * *p*<0.05, ** *p*<0.01.** (B) **Fluorescence intensity of FISH staining forAC006064.4-201 and IF staining of p16INK4a and Mmp3. * *p*<0.05, ** *p*<0.01,*** *p*<0.001.** (C)** Expressionof AC006064.4-201 in HCs after cheating with different concentrations ofDoxorubicin (0 nm/ml, 100nm/ml and 200nm/ml). (*n*=9, 3 donors for three replicates) * *p*<0.05. **Supplementary Figure 2. (A)** QRT-PCR ofAC006064.4-201 in HCs when AC006064.4-201 was knocked down. (*n*=9, 3 donors for three replicates) *** *p*<0.001.** (B)** QRT-PCR ofAC006064.4-201 in HCs when AC006064.4-201 was overexpressed. (*n*=9, 3 donors for three replicates) ** *p*<0.01.** (C) **Western blot ofYBX3, APOBEC3C, PTBP1, LIN28A and IMPDH1 when they were separately knockeddown. **(D) **Knock down efficiency of CDKN1B shRNAs assessed by qRT-PCR. (*n*=9, 3 donors for three replicates) *** *p*<0.001.** (E) **MRNA levels ofMmp3, Mmp13, Sox9, Aggrecan, p16INK4a, p21 and p53 assessed by qRT-PCR whenCDKN1B was knocked down in HCs. (*n*=9,3 donors for three replicates) * *p*<0.05,** *p*<0.01, *** *p*<0.001. **(F) **QRT-PCR of CDKN1Bin HCs when CDKN1B was overexpressed. (*n*=9,3 donors for three replicates) ** *p*<0.01.**(G)** MRNA levels of Mmp3, Mmp13, Sox9, Aggrecan, p16INK4a, p21 and p53assessed by qRT-PCR when AC006064.4-201 was overexpressed or co-overexpressedwith CDKN1B. (*n*=9, 3 donors for threereplicates) * *p*<0.05, ** *p*<0.01, *** *p*<0.001. **Supplementary Figure 3. (A)** Sequence ofAC006064.4-201. (Red sequence, same part as Gm49317-201 sequence) **(B) **Knock down efficiency of Gm49317-201 ASOs assessed by qRT-PCR. (*n*=9, 3 donors for three replicates) ** *p*<0.01. **(C)** Western blot ofPTPB1 in MCs aftering pulled-down with the biotinylated Gm49317-201 mixed probes. **(D) **Knock down efficiency of CDKN1B shRNAs assessed by qRT-PCR. (*n*=9, 3 donors for three replicates) *** *p*<0.001. **Supplementary Figure 4. (A) **Representative images and flurence intensities of IF staining forCDKN1B and Col2a1 in mice cartilage of different groups. (*n*=10) * *p*<0.05, ** *p*<0.01, *** *p*<0.001. Scale bars, 200µm and 50µm. **Supplementary Figure 5. (A)** Representative images and flurence intensities of IF staining forCDKN1B and Col2a1 in mice cartilage of different groups. (*n*=10) * *p*<0.05, ** *p*<0.01. Scale bars, 200µm and 50µm.

**Additional file 2:** **Supplementary Table 1.** RNAseq analysis. **Supplementary Table 2**. RPD-MS analysis. **Supplementary Table 3.** Primer sequences used in this study. **Supplementary Table 4.** Sequences of ASOs, shRNAs and probes. **Supplementary Table 5. **Antibodies usedin this study.

## Data Availability

All data supporting the findings of this study are available within the paper and its supplementary information.

## Abbreviations

OA: Osteoarthritis
LncRNAs: Long non-coding RNAs
PTBP1: Polypyrimidine tract-binding protein 1
CDKN1B: Cyclin-dependent kinase inhibitor 1B
MMTL: Medial meniscotibial ligament
ECM: Extracellular matrix
SnCs: Senescent cells
HCs: Human chondrocytes
MCs: Mouse chondrocytes
qRT-PCR: Quantitative real time PCR
IF: Immunofluorescence
FISH: Fluorescence in situ hybridization
RIP: RNA immunoprecipitation
DMM: Destabilization of the medial meniscus
OARSI: Osteoarthritis Research Society International
RBPs: RNA-binding proteins
