# Supplementary material for: LncRNA AC006064.4–201 serves as a novel molecular marker in alleviating cartilage senescence and protecting against osteoarthritis by destabilizing CDKN1B mRNA via interacting with PTBP1
Source: Biomark Res. 2023 Apr 13;11:39. doi: 10.1186/s40364-023-00477-6 (PMC10099822; doi:10.1186/s40364-023-00477-6)
Supplement: Supplementary file 1 — Additional file 1: Supplementary Figure 1. (A) Expressionof ten lncRNAs in HCs assessed by qRT-PCR after treating with 10ng/ml IL-1β. (n=9, 3 donors for three replicates) * p<0.05, ** p<0.01. (B) Fluorescence intensity of FISH staining forAC006064.4-201 and IF staining of p16INK4a and Mmp3. * p<0.05, ** p<0.01,*** p<0.001. (C) Expressionof AC006064.4-201 in HCs after cheating with different concentrations ofDoxorubicin (0 nm/ml, 100nm/ml and 200nm/ml). (n=9, 3 donors for three replicates) * p<0.05. Supplementary Figure 2. (A) QRT-PCR ofAC006064.4-201 in HCs when AC006064.4-201 was knocked down. (n=9, 3 donors for three replicates) *** p<0.001. (B) QRT-PCR ofAC006064.4-201 in HCs when AC006064.4-201 was overexpressed. (n=9, 3 donors for three replicates) ** p<0.01. (C) Western blot ofYBX3, APOBEC3C, PTBP1, LIN28A and IMPDH1 when they were separately knockeddown. (D) Knock down efficiency of CDKN1B shRNAs assessed by qRT-PCR. (n=9, 3 donors for three replicates) *** p<0.001. (E) MRNA levels ofMmp3, Mmp13, Sox9, Aggrecan, p16INK4a, p21 and p53 assessed by qRT-PCR whenCDKN1B was knocked down in HCs. (n=9,3 donors for three replicates) * p<0.05,** p<0.01, *** p<0.001. (F) QRT-PCR of CDKN1Bin HCs when CDKN1B was overexpressed. (n=9,3 donors for three replicates) ** p<0.01.(G) MRNA levels of Mmp3, Mmp13, Sox9, Aggrecan, p16INK4a, p21 and p53assessed by qRT-PCR when AC006064.4-201 was overexpressed or co-overexpressedwith CDKN1B. (n=9, 3 donors for threereplicates) * p<0.05, ** p<0.01, *** p<0.001. Supplementary Figure 3. (A) Sequence ofAC006064.4-201. (Red sequence, same part as Gm49317-201 sequence) (B) Knock down efficiency of Gm49317-201 ASOs assessed by qRT-PCR. (n=9, 3 donors for three replicates) ** p<0.01. (C) Western blot ofPTPB1 in MCs aftering pulled-down with the biotinylated Gm49317-201 mixed probes. (D) Knock down efficiency of CDKN1B shRNAs assessed by qRT-PCR. (n=9, 3 donors for three replicates) *** p<0.001. Supplementary Figure 4. (A) Representative images [file 40364_2023_477_MOESM1_ESM.docx]

**Supplementary Figures**

Supplementary Figure1


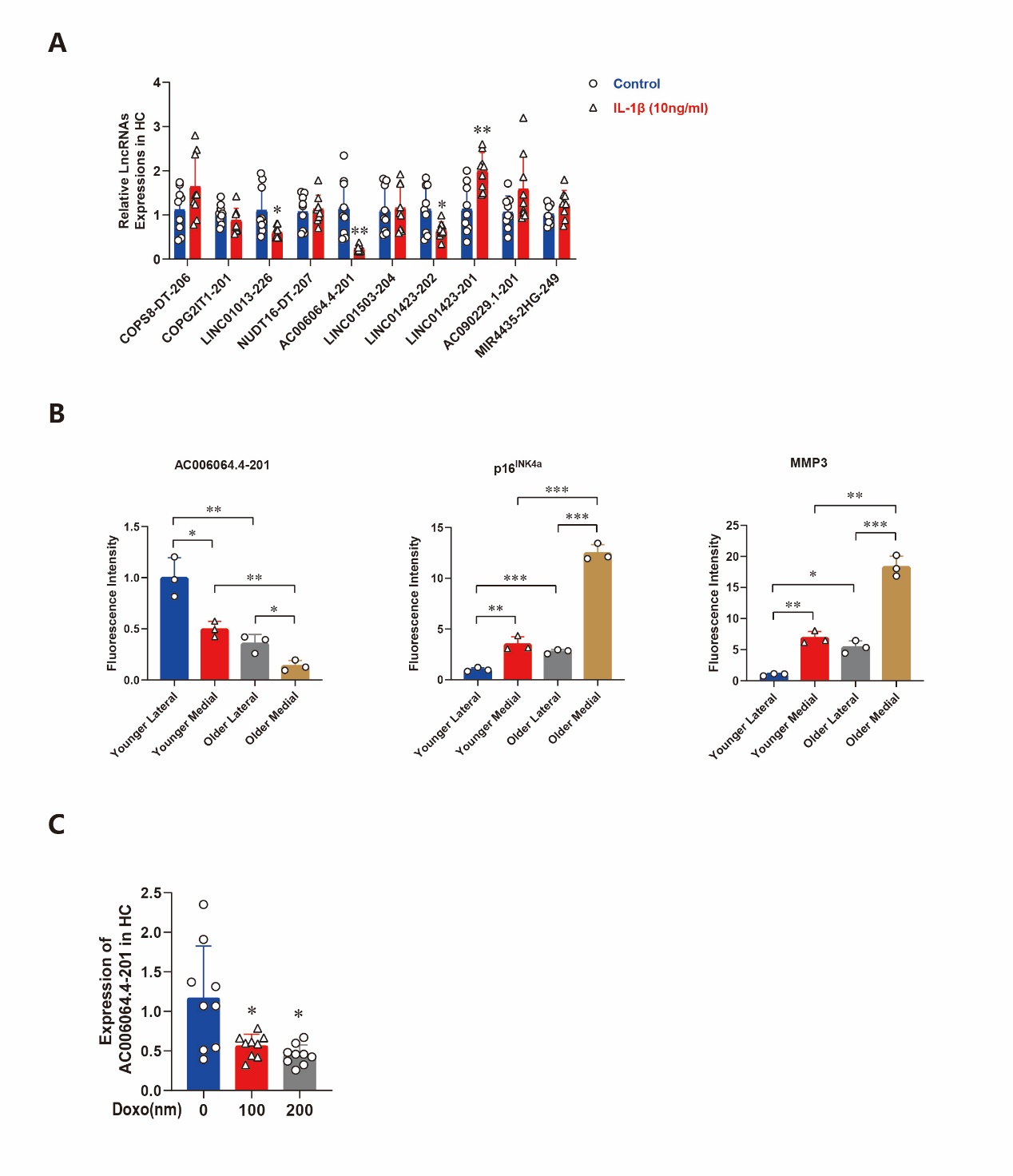


**Supplementary figure1. (A)** Expression of ten lncRNAs in HCs assessed by qRT-PCR after treating with 10ng/ml IL-1β. (n=9, 3 donors for three replicates) * p<0.05, ** p<0.01. **(B)** Fluorescence intensity of FISH staining for AC006064.4-201 and IF staining of p16INK4a and Mmp3. * p<0.05, ** p<0.01, *** p<0.001. **(C)** Expression of AC006064.4-201 in HCs after cheating with different concentrations of Doxorubicin (0 nm/ml, 100nm/ml and 200nm/ml). (n=9, 3 donors for three replicates) * p<0.05.

Supplementary Figure2


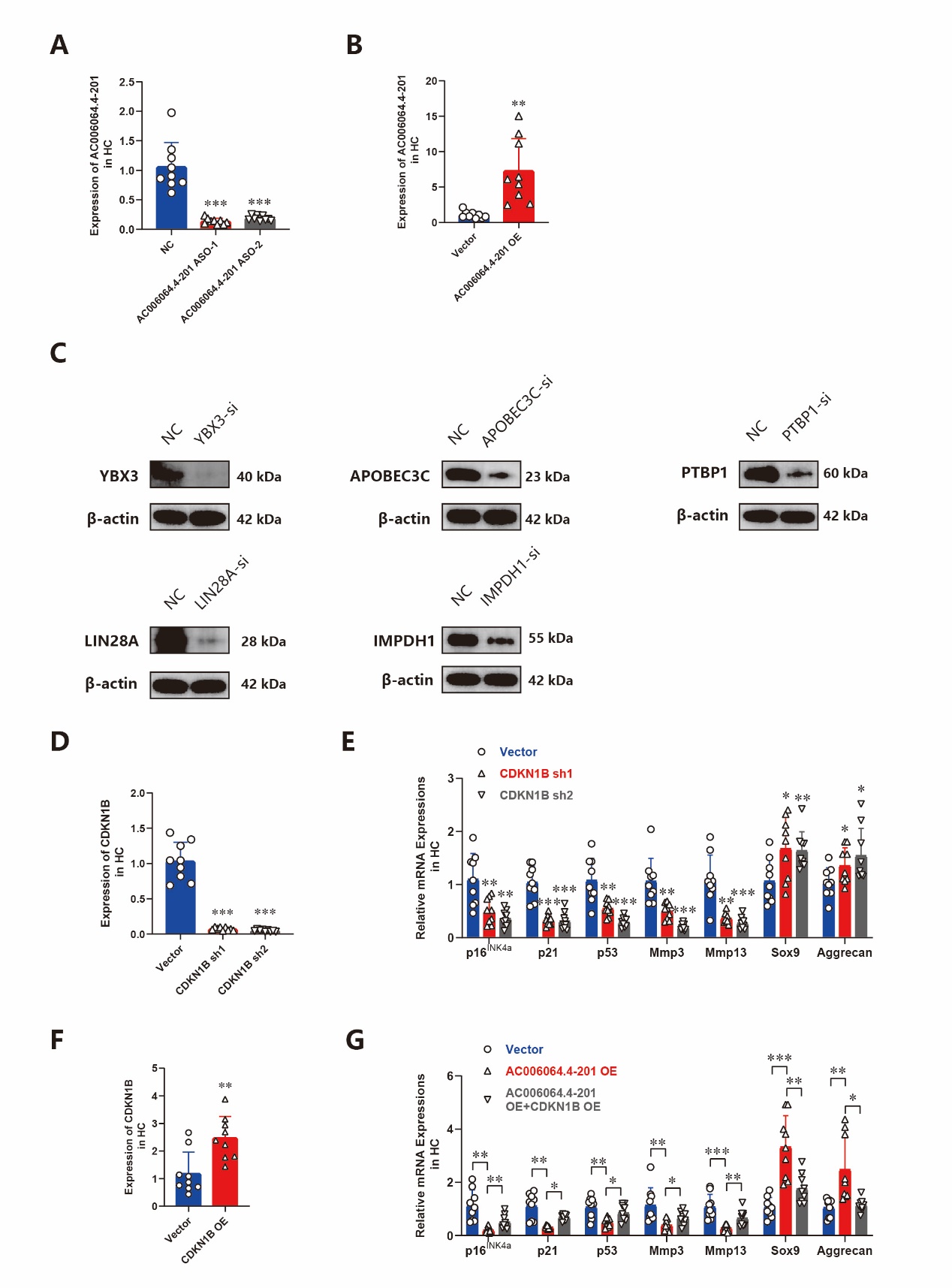


**Supplementary figure2. (A)** QRT-PCR of AC006064.4-201 in HCs when AC006064.4-201 was knocked down. (n=9, 3 donors for three replicates) *** p<0.001. **(B)** QRT-PCR of AC006064.4-201 in HCs when AC006064.4-201 was overexpressed. (n=9, 3 donors for three replicates) ** p<0.01. **(C)** Western blot of YBX3, APOBEC3C, PTBP1, LIN28A and IMPDH1 when they were separately knocked down. **(D)** Knock down efficiency of CDKN1B shRNAs assessed by qRT-PCR. (n=9, 3 donors for three replicates) *** p<0.001. **(E)** MRNA levels of Mmp3, Mmp13, Sox9, Aggrecan, p16INK4a, p21 and p53 assessed by qRT-PCR when CDKN1B was knocked down in HCs. (n=9, 3 donors for three replicates) * p<0.05, ** p<0.01, *** p<0.001. **(F)** QRT-PCR of CDKN1B in HCs when CDKN1B was overexpressed. (n=9, 3 donors for three replicates) ** p<0.01. **(G)** MRNA levels of Mmp3, Mmp13, Sox9, Aggrecan, p16INK4a, p21 and p53 assessed by qRT-PCR when AC006064.4-201 was overexpressed or co-overexpressed with CDKN1B. (n=9, 3 donors for three replicates) * p<0.05, ** p<0.01, *** p<0.001.

Supplementary Figure3


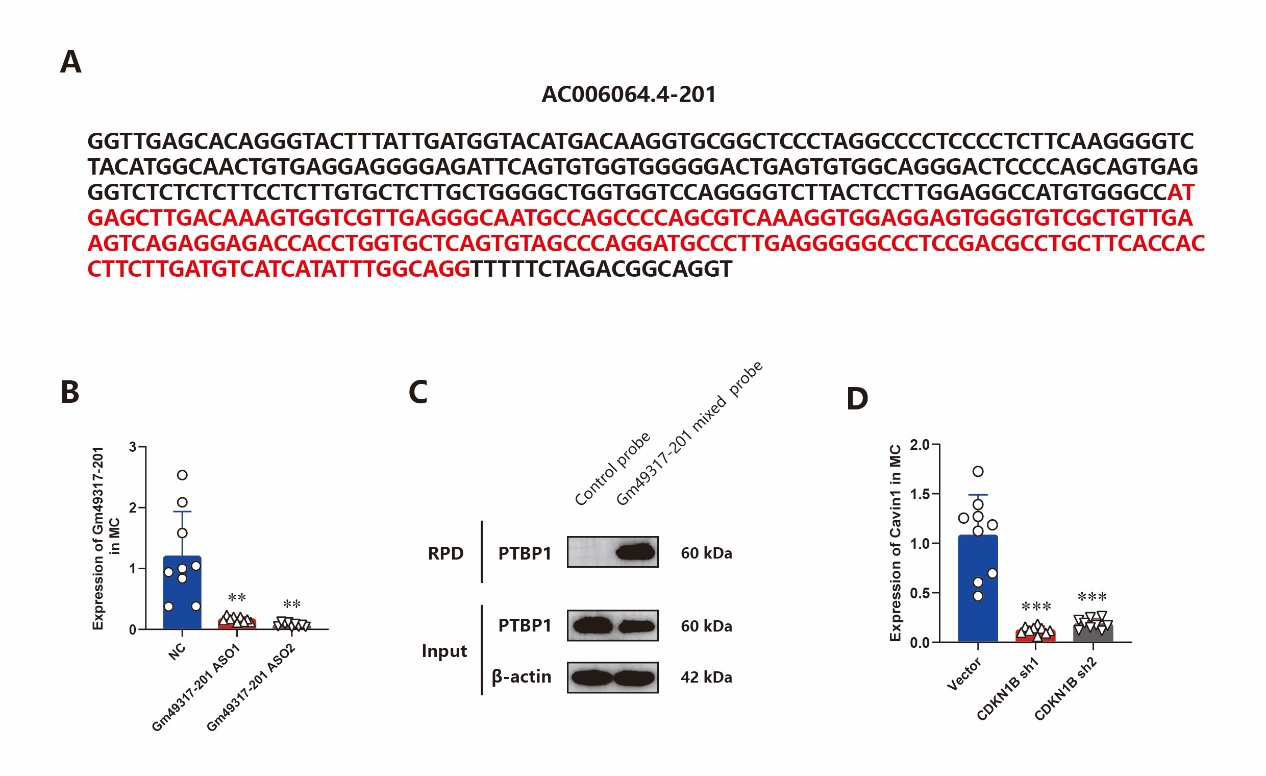


**Supplementary figure3. (A)** Sequence of AC006064.4-201. (Red sequence, same part as Gm49317-201 sequence) **(B)** Knock down efficiency of Gm49317-201 ASOs assessed by qRT-PCR. (n=9, 3 donors for three replicates) ** p<0.01. **(C)** Western blot of PTPB1 in MCs aftering pulled-down with the biotinylated Gm49317-201 mixed probes. **(D)** Knock down efficiency of CDKN1B shRNAs assessed by qRT-PCR. (n=9, 3 donors for three replicates) *** p<0.001.

Supplementary Figure4


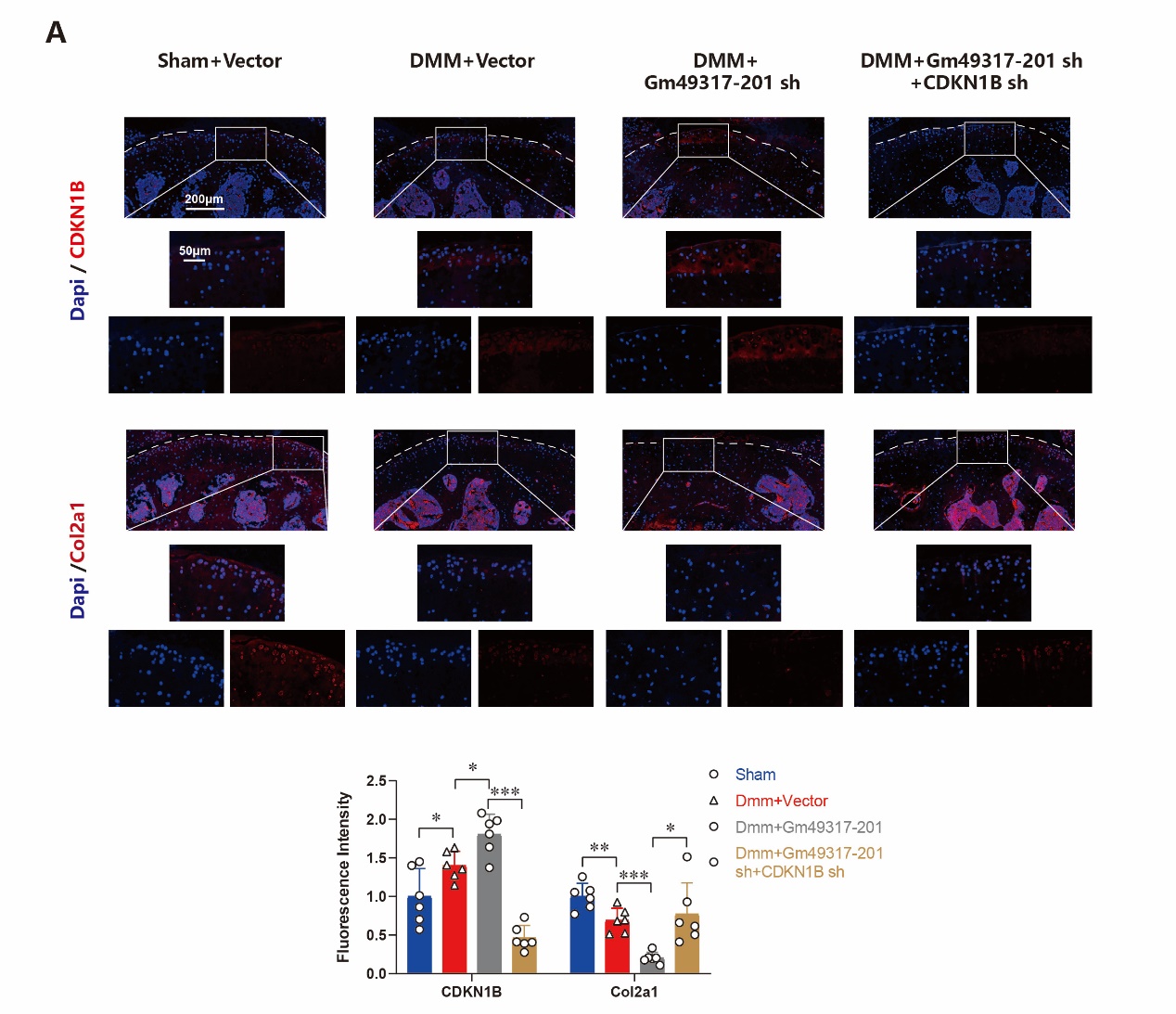


**Supplementary figure4. (A)** Representative images and flurence intensities of IF staining for CDKN1B and Col2a1 in mice cartilage of different groups. (n=10) * p<0.05, ** p<0.01, *** p<0.001. Scale bars, 200µm and 50µm.

Supplementary Figure5


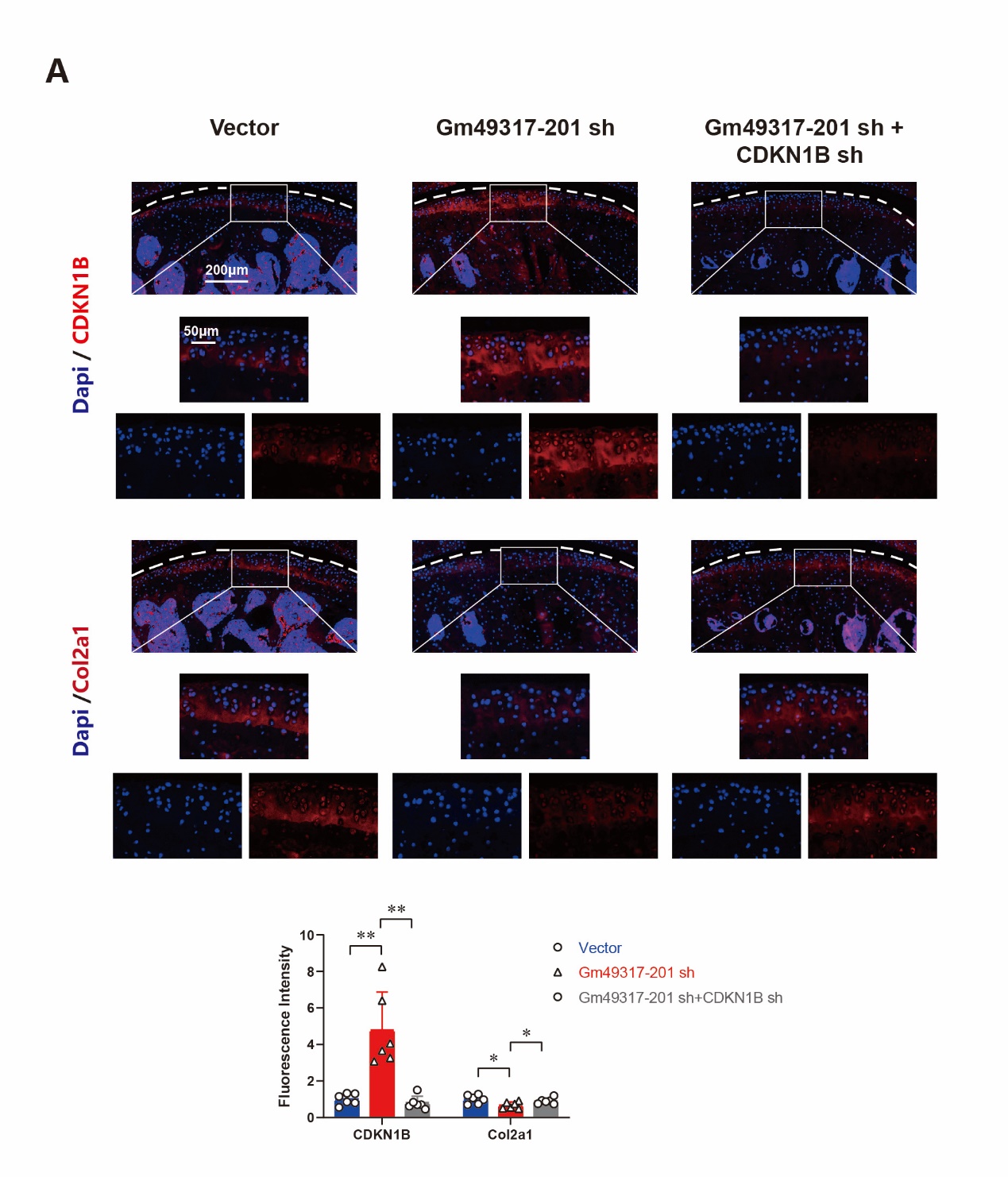


**Supplementary figure5. (A)** Representative images and flurence intensities of IF staining for CDKN1B and Col2a1 in mice cartilage of different groups. (n=10) * p<0.05, ** p<0.01. Scale bars, 200µm and 50µm.
