# Supplementary material for: LncRNA AC006064.4–201 serves as a novel molecular marker in alleviating cartilage senescence and protecting against osteoarthritis by destabilizing CDKN1B mRNA via interacting with PTBP1
Source: Biomark Res. 2023 Apr 13;11:39. doi: 10.1186/s40364-023-00477-6 (PMC10099822; doi:10.1186/s40364-023-00477-6)
Supplement: Supplementary file 2 — Additional file 2: Supplementary Table 1. RNAseq analysis. Supplementary Table 2. RPD-MS analysis. Supplementary Table 3. Primer sequences used in this study. Supplementary Table 4. Sequences of ASOs, shRNAs and probes. Supplementary Table 5. Antibodies usedin this study. [file 40364_2023_477_MOESM2_ESM.docx]

**Tables**

Supplementary Table1 RNAseq analysis

| Transcript id | Log2FC(HCP2/HCP0) | Pvalue | Padjust | Significant | Regulate |
| --- | --- | --- | --- | --- | --- |
| ENST00000431095 | -4.9824 | 1.66E-07 | 1.37E-05 | yes | down |
| ENST00000665401 | -3.70646 | 0.001335 | 0.024992 | yes | down |
| ENST00000651844 | -3.69594 | 9.85E-08 | 8.68E-06 | yes | down |
| ENST00000662166 | -3.61233 | 9.97E-05 | 0.003243 | yes | down |
| ENST00000662294 | -3.40295 | 0.000319 | 0.008117 | yes | down |
| ENST00000602946 | -3.26412 | 0.001024 | 0.02027 | yes | down |
| ENST00000444125 | -3.18787 | 1.38E-06 | 8.56E-05 | yes | down |
| ENST00000436845 | -3.18673 | 2.29E-06 | 0.000134 | yes | down |
| ENST00000414189 | -3.13379 | 1.39E-05 | 0.000619 | yes | down |
| ENST00000590983 | -2.9828 | 5.16E-08 | 5.04E-06 | yes | down |
| ENST00000662635 | -2.92721 | 0.000136 | 0.004129 | yes | down |
| ENST00000561816 | -2.66903 | 0.000373 | 0.009129 | yes | down |
| ENST00000668072 | -2.61995 | 0.002441 | 0.038959 | yes | down |
| ENST00000593078 | -2.61026 | 4.17E-09 | 5.73E-07 | yes | down |
| ENST00000568150 | -2.54729 | 0.00277 | 0.042792 | yes | down |
| ENST00000512817 | -2.53375 | 0.001714 | 0.030315 | yes | down |
| ENST00000604965 | -2.41411 | 1.23E-05 | 0.000563 | yes | down |
| ENST00000663205 | -2.32091 | 0.000989 | 0.019777 | yes | down |
| ENST00000421704 | -2.27809 | 0.0012 | 0.022855 | yes | down |
| ENST00000523301 | -2.27157 | 2.91E-05 | 0.001157 | yes | down |
| ENST00000588925 | -2.24913 | 4.78E-06 | 0.000245 | yes | down |
| ENST00000443303 | -2.21612 | 4.00E-05 | 0.001509 | yes | down |
| ENST00000664579 | -2.21521 | 0.000155 | 0.004615 | yes | down |
| ENST00000463058 | -2.21023 | 0.000704 | 0.015137 | yes | down |
| ENST00000655317 | -2.20554 | 0.001076 | 0.021017 | yes | down |
| ENST00000511699 | -2.18307 | 0.000195 | 0.00546 | yes | down |
| ENST00000655993 | -2.18267 | 0.000247 | 0.006544 | yes | down |
| ENST00000650155 | -2.10313 | 1.24E-06 | 7.83E-05 | yes | down |
| ENST00000653319 | -2.05952 | 0.002973 | 0.045147 | yes | down |
| ENST00000660213 | -1.98417 | 0.001641 | 0.029359 | yes | down |
| ENST00000693348 | -1.96769 | 0.000143 | 0.004286 | yes | down |
| ENST00000666945 | -1.87888 | 0.000453 | 0.010687 | yes | down |
| ENST00000422700 | -1.87429 | 0.000292 | 0.007598 | yes | down |
| ENST00000485055 | -1.83843 | 0.001416 | 0.026152 | yes | down |
| ENST00000616822 | -1.81473 | 2.40E-05 | 0.000986 | yes | down |
| ENST00000441722 | -1.81452 | 0.000119 | 0.003716 | yes | down |
| ENST00000657484 | -1.76105 | 0.001336 | 0.024992 | yes | down |
| ENST00000620791 | -1.74632 | 0.001998 | 0.033786 | yes | down |
| ENST00000654880 | -1.65539 | 0.003158 | 0.046858 | yes | down |
| ENST00000610128 | -1.65102 | 1.38E-05 | 0.000615 | yes | down |
| ENST00000693158 | -1.65039 | 0.000747 | 0.015883 | yes | down |
| ENST00000375640 | -1.6233 | 2.72E-07 | 2.09E-05 | yes | down |
| ENST00000660194 | -1.61478 | 0.001711 | 0.030297 | yes | down |
| ENST00000433843 | -1.59017 | 0.000807 | 0.016961 | yes | down |
| ENST00000517335 | -1.58536 | 0.000526 | 0.012052 | yes | down |
| ENST00000670617 | -1.54175 | 6.81E-05 | 0.002391 | yes | down |
| ENST00000570269 | -1.51315 | 1.94E-07 | 1.56E-05 | yes | down |
| ENST00000420877 | -1.49934 | 3.19E-05 | 0.001249 | yes | down |
| ENST00000478845 | -1.46491 | 0.001899 | 0.032639 | yes | down |
| ENST00000656432 | -1.44819 | 0.000583 | 0.013001 | yes | down |
| ENST00000375635 | -1.44225 | 0.000446 | 0.010585 | yes | down |
| ENST00000568302 | -1.43605 | 7.04E-05 | 0.002456 | yes | down |
| ENST00000688805 | -1.43457 | 4.55E-07 | 3.30E-05 | yes | down |
| ENST00000670682 | -1.42314 | 0.003433 | 0.04974 | yes | down |
| ENST00000375638 | -1.41914 | 3.03E-06 | 0.000169 | yes | down |
| ENST00000564670 | -1.4084 | 0.003188 | 0.047157 | yes | down |
| ENST00000562038 | -1.40443 | 2.52E-07 | 1.97E-05 | yes | down |
| ENST00000667979 | -1.40149 | 1.93E-06 | 0.000115 | yes | down |
| ENST00000642367 | -1.37588 | 1.48E-06 | 9.13E-05 | yes | down |
| ENST00000580180 | -1.36108 | 8.07E-07 | 5.37E-05 | yes | down |
| ENST00000547717 | -1.31903 | 1.42E-05 | 0.000631 | yes | down |
| ENST00000475947 | -1.31819 | 1.24E-05 | 0.000568 | yes | down |
| ENST00000422304 | -1.29793 | 5.69E-06 | 0.000284 | yes | down |
| ENST00000605056 | -1.24295 | 0.001941 | 0.03322 | yes | down |
| ENST00000671650 | -1.18734 | 0.001032 | 0.020407 | yes | down |
| ENST00000667533 | -1.18653 | 0.00341 | 0.049644 | yes | down |
| ENST00000643276 | -1.18573 | 0.000949 | 0.019162 | yes | down |
| ENST00000455485 | -1.16502 | 0.000164 | 0.00482 | yes | down |
| ENST00000620266 | -1.15097 | 0.000464 | 0.010896 | yes | down |
| ENST00000505448 | -1.14295 | 0.001118 | 0.021647 | yes | down |
| ENST00000602529 | -1.13158 | 0.002981 | 0.045177 | yes | down |
| ENST00000625013 | -1.12395 | 0.003426 | 0.049697 | yes | down |
| ENST00000685861 | -1.11442 | 6.19E-05 | 0.002215 | yes | down |
| ENST00000481027 | -1.09836 | 0.00064 | 0.014037 | yes | down |
| ENST00000650609 | -1.09393 | 0.000244 | 0.006479 | yes | down |
| ENST00000451424 | -1.09304 | 2.63E-05 | 0.00106 | yes | down |
| ENST00000564352 | -1.03728 | 0.002977 | 0.045156 | yes | down |
| ENST00000483140 | -1.031 | 0.003106 | 0.046331 | yes | down |
| ENST00000663781 | -1.00446 | 0.000368 | 0.009066 | yes | down |
| ENST00000532315 | 1.014388 | 0.0022 | 0.036275 | yes | up |
| ENST00000337752 | 1.034955 | 5.12E-05 | 0.001877 | yes | up |
| ENST00000687367 | 1.077065 | 0.003034 | 0.045647 | yes | up |
| ENST00000689405 | 1.103426 | 0.001243 | 0.023562 | yes | up |
| ENST00000468186 | 1.130213 | 0.000167 | 0.004861 | yes | up |
| ENST00000602461 | 1.172404 | 1.86E-05 | 0.000792 | yes | up |
| ENST00000606008 | 1.196803 | 0.00171 | 0.030297 | yes | up |
| ENST00000652782 | 1.25576 | 0.002351 | 0.037947 | yes | up |
| ENST00000413987 | 1.266732 | 9.74E-06 | 0.000459 | yes | up |
| ENST00000602502 | 1.268575 | 0.001732 | 0.03047 | yes | up |
| ENST00000481368 | 1.299725 | 9.59E-05 | 0.003171 | yes | up |
| ENST00000421331 | 1.332184 | 0.001359 | 0.025284 | yes | up |
| ENST00000617468 | 1.338344 | 0.001884 | 0.032484 | yes | up |
| ENST00000660431 | 1.361556 | 0.003144 | 0.046677 | yes | up |
| ENST00000623655 | 1.424809 | 0.000988 | 0.01977 | yes | up |
| ENST00000608721 | 1.493593 | 0.000204 | 0.005657 | yes | up |
| ENST00000584934 | 1.503875 | 0.0007 | 0.01508 | yes | up |
| ENST00000692517 | 1.504645 | 3.57E-05 | 0.001369 | yes | up |
| ENST00000453135 | 1.551602 | 0.001867 | 0.03224 | yes | up |
| ENST00000664908 | 1.562475 | 0.000635 | 0.013957 | yes | up |
| ENST00000606070 | 1.576744 | 2.03E-05 | 0.00085 | yes | up |
| ENST00000602478 | 1.585233 | 2.53E-07 | 1.97E-05 | yes | up |
| ENST00000647812 | 1.591652 | 0.000295 | 0.007654 | yes | up |
| ENST00000610494 | 1.65576 | 0.00036 | 0.008909 | yes | up |
| ENST00000688135 | 1.675893 | 0.002879 | 0.04409 | yes | up |
| ENST00000666376 | 1.700042 | 0.003441 | 0.049806 | yes | up |
| ENST00000664186 | 1.727637 | 3.40E-08 | 3.58E-06 | yes | up |
| ENST00000658479 | 1.737904 | 0.000156 | 0.004641 | yes | up |
| ENST00000563601 | 1.747541 | 0.000327 | 0.008304 | yes | up |
| ENST00000518947 | 1.756187 | 0.003342 | 0.048909 | yes | up |
| ENST00000653771 | 1.758515 | 0.000179 | 0.005114 | yes | up |
| ENST00000618589 | 1.781027 | 0.000547 | 0.012379 | yes | up |
| ENST00000650796 | 1.810483 | 4.97E-08 | 4.89E-06 | yes | up |
| ENST00000689375 | 1.813397 | 0.001003 | 0.019949 | yes | up |
| ENST00000567359 | 1.817168 | 0.000604 | 0.01339 | yes | up |
| ENST00000666085 | 1.828138 | 0.00017 | 0.004926 | yes | up |
| ENST00000656793 | 1.831906 | 0.000828 | 0.017301 | yes | up |
| ENST00000688102 | 1.856979 | 0.001803 | 0.031396 | yes | up |
| ENST00000629145 | 1.877239 | 0.000376 | 0.009171 | yes | up |
| ENST00000623543 | 1.879195 | 9.39E-06 | 0.000449 | yes | up |
| ENST00000537869 | 1.891794 | 0.003076 | 0.046001 | yes | up |
| ENST00000619666 | 1.892163 | 0.000319 | 0.008117 | yes | up |
| ENST00000461448 | 1.90045 | 0.000132 | 0.004023 | yes | up |
| ENST00000645232 | 1.90971 | 0.002343 | 0.037902 | yes | up |
| ENST00000620246 | 1.918363 | 3.38E-05 | 0.001313 | yes | up |
| ENST00000653192 | 1.939101 | 0.002272 | 0.037167 | yes | up |
| ENST00000621230 | 1.942063 | 9.17E-08 | 8.25E-06 | yes | up |
| ENST00000676450 | 1.953863 | 6.46E-05 | 0.002299 | yes | up |
| ENST00000563592 | 1.958008 | 0.002327 | 0.037671 | yes | up |
| ENST00000569710 | 1.962937 | 8.59E-05 | 0.002918 | yes | up |
| ENST00000689482 | 1.964704 | 0.001455 | 0.026687 | yes | up |
| ENST00000586076 | 1.968215 | 0.001967 | 0.033503 | yes | up |
| ENST00000618151 | 1.969963 | 0.000263 | 0.006908 | yes | up |
| ENST00000602919 | 2.012448 | 6.49E-05 | 0.002303 | yes | up |
| ENST00000668968 | 2.012472 | 0.000542 | 0.012289 | yes | up |
| ENST00000620019 | 2.037363 | 3.77E-05 | 0.001435 | yes | up |
| ENST00000538868 | 2.06811 | 2.47E-05 | 0.001007 | yes | up |
| ENST00000474814 | 2.087705 | 6.46E-08 | 6.09E-06 | yes | up |
| ENST00000607957 | 2.106727 | 2.69E-05 | 0.001082 | yes | up |
| ENST00000603994 | 2.11423 | 0.00184 | 0.031919 | yes | up |
| ENST00000663389 | 2.124382 | 0.000191 | 0.005381 | yes | up |
| ENST00000453837 | 2.132497 | 0.001618 | 0.029025 | yes | up |
| ENST00000688375 | 2.141819 | 0.000431 | 0.010286 | yes | up |
| ENST00000561909 | 2.155622 | 0.002288 | 0.037318 | yes | up |
| ENST00000432045 | 2.157372 | 1.33E-12 | 3.94E-10 | yes | up |
| ENST00000689147 | 2.158702 | 0.000176 | 0.00507 | yes | up |
| ENST00000654855 | 2.160689 | 0.000911 | 0.018607 | yes | up |
| ENST00000689900 | 2.162747 | 0.000204 | 0.005652 | yes | up |
| ENST00000608917 | 2.184013 | 0.00012 | 0.003729 | yes | up |
| ENST00000569378 | 2.196793 | 0.00327 | 0.048144 | yes | up |
| ENST00000650239 | 2.198182 | 0.000831 | 0.017334 | yes | up |
| ENST00000576808 | 2.199771 | 0.001429 | 0.026329 | yes | up |
| ENST00000602507 | 2.21213 | 0.003211 | 0.047423 | yes | up |
| ENST00000567209 | 2.233243 | 0.002505 | 0.039624 | yes | up |
| ENST00000564152 | 2.245294 | 6.40E-07 | 4.43E-05 | yes | up |
| ENST00000635591 | 2.261451 | 3.41E-06 | 0.000185 | yes | up |
| ENST00000654107 | 2.267618 | 4.51E-05 | 0.001669 | yes | up |
| ENST00000655771 | 2.269217 | 0.001978 | 0.03356 | yes | up |
| ENST00000565695 | 2.291617 | 1.24E-06 | 7.83E-05 | yes | up |
| ENST00000609998 | 2.293756 | 0.001769 | 0.030956 | yes | up |
| ENST00000429998 | 2.296555 | 0.000117 | 0.003668 | yes | up |
| ENST00000615251 | 2.309666 | 2.45E-06 | 0.000142 | yes | up |
| ENST00000692406 | 2.31406 | 0.000247 | 0.006553 | yes | up |
| ENST00000666785 | 2.323105 | 0.000373 | 0.009128 | yes | up |
| ENST00000531559 | 2.325529 | 0.002763 | 0.042706 | yes | up |
| ENST00000564287 | 2.335927 | 0.000161 | 0.004743 | yes | up |
| ENST00000658007 | 2.344029 | 0.001199 | 0.022855 | yes | up |
| ENST00000612330 | 2.345059 | 0.000166 | 0.004861 | yes | up |
| ENST00000690542 | 2.345759 | 9.23E-05 | 0.003074 | yes | up |
| ENST00000608783 | 2.372583 | 1.55E-05 | 0.000678 | yes | up |
| ENST00000432521 | 2.394756 | 0.001145 | 0.022073 | yes | up |
| ENST00000605386 | 2.40131 | 4.09E-05 | 0.001539 | yes | up |
| ENST00000611877 | 2.409524 | 3.20E-06 | 0.000175 | yes | up |
| ENST00000670012 | 2.445255 | 0.001371 | 0.025404 | yes | up |
| ENST00000666601 | 2.45132 | 0.001847 | 0.031989 | yes | up |
| ENST00000691294 | 2.469803 | 3.51E-06 | 0.00019 | yes | up |
| ENST00000500698 | 2.485682 | 6.32E-10 | 9.83E-08 | yes | up |
| ENST00000670650 | 2.533698 | 6.33E-07 | 4.39E-05 | yes | up |
| ENST00000692840 | 2.542296 | 1.82E-05 | 0.000778 | yes | up |
| ENST00000422679 | 2.552767 | 0.000129 | 0.003961 | yes | up |
| ENST00000656268 | 2.556879 | 4.88E-08 | 4.83E-06 | yes | up |
| ENST00000471537 | 2.562266 | 0.000686 | 0.014813 | yes | up |
| ENST00000655830 | 2.571711 | 0.000573 | 0.012836 | yes | up |
| ENST00000572856 | 2.605805 | 1.04E-15 | 5.86E-13 | yes | up |
| ENST00000534271 | 2.611068 | 9.45E-06 | 0.00045 | yes | up |
| ENST00000668300 | 2.628823 | 2.88E-09 | 3.98E-07 | yes | up |
| ENST00000557989 | 2.633921 | 0.000743 | 0.015827 | yes | up |
| ENST00000392097 | 2.638313 | 5.87E-13 | 1.89E-10 | yes | up |
| ENST00000531126 | 2.650316 | 0.001104 | 0.021418 | yes | up |
| ENST00000425653 | 2.659995 | 0.000181 | 0.00515 | yes | up |
| ENST00000689770 | 2.665267 | 2.41E-05 | 0.000986 | yes | up |
| ENST00000538654 | 2.672722 | 0.000227 | 0.006116 | yes | up |
| ENST00000659015 | 2.683927 | 0.000572 | 0.012836 | yes | up |
| ENST00000603468 | 2.68869 | 6.33E-12 | 1.52E-09 | yes | up |
| ENST00000564925 | 2.690052 | 6.06E-05 | 0.002176 | yes | up |
| ENST00000623664 | 2.69367 | 9.54E-06 | 0.000452 | yes | up |
| ENST00000612566 | 2.705155 | 2.78E-07 | 2.13E-05 | yes | up |
| ENST00000427278 | 2.732083 | 4.51E-05 | 0.001669 | yes | up |
| ENST00000641308 | 2.739879 | 0.003117 | 0.046436 | yes | up |
| ENST00000567598 | 2.745623 | 3.01E-10 | 5.14E-08 | yes | up |
| ENST00000692264 | 2.756615 | 2.00E-05 | 0.000842 | yes | up |
| ENST00000670963 | 2.783161 | 1.68E-05 | 0.000728 | yes | up |
| ENST00000664307 | 2.816774 | 4.00E-07 | 2.94E-05 | yes | up |
| ENST00000382641 | 2.816916 | 1.09E-05 | 0.000509 | yes | up |
| ENST00000434401 | 2.818323 | 1.29E-05 | 0.000586 | yes | up |
| ENST00000414046 | 2.829647 | 1.49E-05 | 0.000655 | yes | up |
| ENST00000660312 | 2.842935 | 9.81E-05 | 0.003223 | yes | up |
| ENST00000422008 | 2.843015 | 0.002298 | 0.037446 | yes | up |
| ENST00000691255 | 2.843937 | 1.94E-06 | 0.000115 | yes | up |
| ENST00000566297 | 2.849871 | 0.000362 | 0.008949 | yes | up |
| ENST00000609281 | 2.879644 | 1.55E-06 | 9.54E-05 | yes | up |
| ENST00000613093 | 2.901214 | 5.65E-12 | 1.38E-09 | yes | up |
| ENST00000434112 | 2.903879 | 0.000763 | 0.016187 | yes | up |
| ENST00000664871 | 2.938894 | 0.003069 | 0.045928 | yes | up |
| ENST00000418602 | 2.949588 | 0.001079 | 0.02105 | yes | up |
| ENST00000658856 | 2.975608 | 6.58E-05 | 0.002325 | yes | up |
| ENST00000581556 | 2.985091 | 1.55E-07 | 1.29E-05 | yes | up |
| ENST00000577781 | 2.998829 | 4.18E-06 | 0.00022 | yes | up |
| ENST00000686140 | 3.048027 | 1.35E-05 | 0.000606 | yes | up |
| ENST00000607044 | 3.05331 | 6.96E-05 | 0.002438 | yes | up |
| ENST00000554254 | 3.054519 | 1.79E-05 | 0.000769 | yes | up |
| ENST00000330539 | 3.082913 | 0.000139 | 0.004209 | yes | up |
| ENST00000606274 | 3.086019 | 4.45E-06 | 0.000231 | yes | up |
| ENST00000415386 | 3.111669 | 6.29E-05 | 0.002248 | yes | up |
| ENST00000569473 | 3.111777 | 5.30E-12 | 1.33E-09 | yes | up |
| ENST00000681370 | 3.120375 | 0.001533 | 0.027838 | yes | up |
| ENST00000685356 | 3.139067 | 5.17E-07 | 3.66E-05 | yes | up |
| ENST00000658363 | 3.143089 | 9.62E-05 | 0.003178 | yes | up |
| ENST00000669966 | 3.161324 | 0.000308 | 0.007882 | yes | up |
| ENST00000651492 | 3.198732 | 3.13E-06 | 0.000172 | yes | up |
| ENST00000685150 | 3.208003 | 0.00011 | 0.003515 | yes | up |
| ENST00000623136 | 3.212997 | 3.19E-05 | 0.001249 | yes | up |
| ENST00000505564 | 3.232588 | 0.000195 | 0.00546 | yes | up |
| ENST00000563105 | 3.236459 | 0.000336 | 0.008478 | yes | up |
| ENST00000566372 | 3.245744 | 2.50E-09 | 3.50E-07 | yes | up |
| ENST00000573866 | 3.259306 | 1.45E-11 | 3.24E-09 | yes | up |
| ENST00000605834 | 3.281111 | 5.93E-11 | 1.19E-08 | yes | up |
| ENST00000665295 | 3.352277 | 7.59E-08 | 7.07E-06 | yes | up |
| ENST00000561318 | 3.35375 | 0.002324 | 0.037671 | yes | up |
| ENST00000415067 | 3.355569 | 2.62E-05 | 0.00106 | yes | up |
| ENST00000521487 | 3.436167 | 7.97E-06 | 0.000386 | yes | up |
| ENST00000599904 | 3.472667 | 2.42E-05 | 0.000989 | yes | up |
| ENST00000648705 | 3.48514 | 0.000501 | 0.011591 | yes | up |
| ENST00000685830 | 3.534626 | 7.19E-07 | 4.89E-05 | yes | up |
| ENST00000541391 | 3.556731 | 2.14E-08 | 2.39E-06 | yes | up |
| ENST00000666735 | 3.684448 | 3.99E-11 | 8.25E-09 | yes | up |
| ENST00000566847 | 3.688509 | 0.000467 | 0.010952 | yes | up |
| ENST00000441356 | 3.690616 | 6.24E-07 | 4.34E-05 | yes | up |
| ENST00000689263 | 3.779585 | 1.56E-06 | 9.57E-05 | yes | up |
| ENST00000538062 | 3.818718 | 0.001676 | 0.029827 | yes | up |
| ENST00000412276 | 3.823889 | 5.59E-05 | 0.002032 | yes | up |
| ENST00000658405 | 3.988989 | 6.50E-07 | 4.48E-05 | yes | up |
| ENST00000606034 | 4.307395 | 1.69E-15 | 9.28E-13 | yes | up |
| ENST00000663804 | 4.319352 | 4.16E-13 | 1.44E-10 | yes | up |
| ENST00000652364 | 4.442661 | 5.35E-06 | 0.000271 | yes | up |
| ENST00000613639 | 4.61564 | 1.82E-14 | 8.56E-12 | yes | up |
| ENST00000691200 | 4.649863 | 2.66E-08 | 2.87E-06 | yes | up |
| ENST00000432377 | 4.71102 | 2.81E-17 | 2.04E-14 | yes | up |
| ENST00000686810 | 5.045357 | 2.30E-12 | 6.24E-10 | yes | up |
| ENST00000415237 | 5.20321 | 6.72E-15 | 3.53E-12 | yes | up |

Supplementary Table2 RPD-MS analysis

| Gene names | LFQ intensity A1  (Control probe) | LFQ intensity A2  (AC006064.4-201 probe) | Score |
| --- | --- | --- | --- |
| YBX3 | 0 | 66992000 | 228.84 |
| APOBEC3C | 0 | 565500000 | 199.12 |
| PTBP1 | 0 | 6807600 | 184.49 |
| LIN28A | 0 | 55081000 | 180.18 |
| IMPDH1 | 0 | 21250000 | 170.87 |
| PCCB | 0 | 739590000 | 145.89 |
| SUGP2 | 0 | 19526000 | 140.16 |
| SSR4 | 0 | 46409000 | 127.4 |
| SRSF3 | 0 | 375010000 | 122.79 |
| CETN2 | 0 | 11699000 | 121.28 |
| TARDBP | 0 | 6108800 | 117.4 |
| FABP5 | 0 | 4324800 | 112.15 |
| CCDC12 | 0 | 13418000 | 106.75 |
| GCN1L1 | 0 | 3313500 | 105.99 |
| RALY | 0 | 7767200 | 105.52 |
| RNPC3 | 0 | 34346000 | 105.4 |
| IMMT | 0 | 5912600 | 105.31 |
| LOR | 0 | 2083200 | 105.17 |
| NDUFS3 | 0 | 6364700 | 101.95 |
| RPL30 | 0 | 8747700 | 100.55 |
| ERLIN1 | 0 | 3324400 | 100.48 |
| PDF | 0 | 3531000 | 98.943 |
| SSR3 | 0 | 8150700 | 96.103 |
| MCU | 0 | 6404100 | 96.059 |
| PSMD14 | 0 | 5941800 | 94.297 |
| RPL24 | 0 | 28600000 | 94.297 |
| ECH1 | 0 | 7567600 | 91.03 |
| EMG1 | 0 | 4043800 | 89.247 |
| ATP5L | 0 | 7375600 | 89.08 |
| FARSA | 0 | 13687000 | 88.681 |
| LIN28B | 0 | 28485000 | 87.639 |
| NR1H2;NR1H3 | 0 | 8299300 | 87.498 |
| FUBP3 | 0 | 12318000 | 86.467 |
| NOP16 | 0 | 4275300 | 85.958 |
| C14orf166 | 0 | 8216000 | 85.212 |
| RBMS3 | 0 | 110810000 | 84.658 |
| RPS13 | 0 | 2114400 | 82.452 |
| PKP1 | 0 | 6255100 | 81.865 |
| TMED9 | 0 | 23090000 | 81.548 |
| DBT | 0 | 259560000 | 81.278 |
| ANKZF1 | 0 | 3406200 | 80.763 |
| TUBB2A;TUBB2B | 0 | 19404000 | 80.763 |
| RBMS2 | 0 | 50031000 | 80.24 |
| NDUFA13 | 0 | 7099200 | 79.906 |
| TRAF6 | 0 | 44351000 | 78.763 |
| TSR1 | 0 | 6505800 | 78.653 |
| TIAL1 | 0 | 57604000 | 77.379 |
| HSD17B12 | 0 | 2359500 | 75.294 |
| CHTOP | 0 | 32368000 | 74.944 |
| CAND1 | 0 | 6632000 | 73.781 |
| QARS | 0 | 7952600 | 73.26 |
| EP300;CREBBP | 0 | 3563100 | 72.879 |
| SYNCRIP | 0 | 7067600 | 72.643 |
| KARS | 0 | 3644300 | 72.234 |
| PGM3 | 0 | 12504000 | 71.342 |
| SF3B4 | 0 | 148920000 | 70.525 |
| CTR9 | 0 | 8178500 | 70.1 |
| RPL36AL;RPL36A | 0 | 20436000 | 69.825 |
| RNF2;RING1 | 0 | 9002800 | 69.045 |
| EEF2 | 0 | 12858000 | 69.01 |
| RPS15A | 0 | 6725000 | 68.809 |
| MAD2L1 | 0 | 24017000 | 67.897 |
| DLAT | 0 | 4347400 | 67.776 |
| SLC25A24 | 0 | 6566300 | 67.726 |
| TOMM40 | 0 | 8830300 | 67.563 |
| AP2S1 | 0 | 22483000 | 67.169 |
| RNH1 | 0 | 6766800 | 67.022 |
| RWDD4 | 0 | 3831200 | 66.621 |
| PSMC1 | 0 | 4318000 | 65.563 |
| NCLN | 0 | 4161900 | 65.305 |
| ARCN1 | 0 | 15111000 | 65.252 |
| PDLIM5 | 0 | 22679000 | 64.297 |
| RPL22 | 0 | 20062000 | 60.641 |
| ATP5C1 | 0 | 4761500 | 60.518 |
| MCM4 | 0 | 17047000 | 59.542 |
| MTHFD1 | 0 | 10546000 | 58.978 |
| AXL | 0 | 3202600 | 56.258 |
| IPO5 | 0 | 11896000 | 55.549 |
| XPOT | 0 | 3563700 | 55.37 |
| XP32 | 0 | 9521100 | 54.982 |
| PTBP3 | 0 | 26706000 | 54.776 |
| APOBEC3B | 0 | 30221000 | 54.539 |
| EPRS | 0 | 1939300 | 54.259 |
| DPM1 | 0 | 3239200 | 54.143 |
| DDX19A;DDX19B | 0 | 11433000 | 53.779 |
| RPS4X | 0 | 32741000 | 52.344 |
| PYCR1;PYCR2 | 0 | 8840300 | 51.092 |
| PDIA6 | 0 | 3519700 | 50.284 |
| POLD1 | 0 | 2307000 | 49.715 |
| PPIE | 0 | 22226000 | 49.097 |
| RIC8A | 0 | 5230300 | 48.656 |
| UQCRC1 | 0 | 6202200 | 46.663 |
| FLNC;FLNB | 0 | 10953000 | 46.481 |
| MTCH2 | 0 | 21596000 | 44.426 |
| CDC42 | 0 | 32608000 | 43.454 |
| PPM1G | 0 | 3891400 | 42.556 |
| AZGP1 | 0 | 5855000 | 38.634 |
| SMC2 | 0 | 8398300 | 36.847 |
| LRP1 | 0 | 8461900 | 36.525 |
| DDX1 | 0 | 4244900 | 29.544 |

Supplementary Table3 Primer sequences used in this study

| Primer set | Gene ID | Primers | Sequences |
| --- | --- | --- | --- |
| LncRNA (human)  COPS8-DT-206 |  | Forward  Reverse | GCTGTTGCTTCACATGTCATC  CCTTAGGGAAAGGCCGTCATC |
| LncRNA (human)  COPG2IT1-201 |  | Forward  Reverse | GGCAGGACCTGTGTAAGAAGA  AAACTCTACAGCCAGTCCCG |
| LncRNA (human)  LINC01013-226 |  | Forward  Reverse | CAGGACACCCACATGACTAGG  GCTGATGCAGTCACAGGTTC |
| LncRNA (human)  NUDT16-DT-207 |  | Forward  Reverse | CCAGGAACCTTAGGGAAACCATA  GATTCTGACGTAGGCGTTTATGTA |
| LncRNA (human)  AC006064.4-201 |  | Forward  Reverse | CATGTGGGCCATGAGCTTG  GCCGTCTAGAAAAACCTGCC |
| LncRNA (human)  LINC01503-204 |  | Forward  Reverse | GGGACGGAGACAAATGACGG  CCTGACACGTAGGTACACACT |
| LncRNA (human)  LINC01423-202 |  | Forward  Reverse | ACTCTCTTTTAAACAGGACCCTCC  TCATTGCCTTCATCAACCTTCTG |
| LncRNA (human)  LINC01423-201 |  | Forward  Reverse | GTCCACCGGTGAAAAGGAGA  TCATTGCCTTCATCAACCTTCTG |
| LncRNA (human)  AC090229.1-201 |  | Forward  Reverse | ACCACAAACACAGAGGTCGAA  TTGGCTCTAGAAACAGCCCT |
| LncRNA (human)  MIR4435-2HG-249 |  | Forward  Reverse | ACAAGCAAAGTGGATCAGCA  AGGAGCAGAGCAGGGAAAAAT |
| ACTB  (human) | 60 | Forward  Reverse | AGAGCTACGAGCTGCCTGAC  AGCACTGTGTTGGCGTACAG |
| GAPDH  (human) | 2597 | Forward  Reverse | AGCCACATCGCTCAGACAC  GCCCAATACGACCAAATCC |
| Mmp3  (human) | 4314 | Forward  Reverse | CCTACAAGGAGGCAGGCAAG  CCCGTCACCTCCAATCCAAG |
| Mmp13  (human) | 4322 | Forward  Reverse | TCGGCCACTCCTTAGGTCTT  AAGTGGCTTTTGCCGGTGTA |
| Sox9  (human) | 6662 | Forward  Reverse | GCTCTGGAGACTTCTGAACGA  CCGTTCTTCACCGACTTCCT |
| Aggrecan  (human)\ | 176 | Forward  Reverse | ACTCTGGGTTTTCGTGACTCT  ACACTCAGCGAGTTGTCATGG |
| P16^INK4a^  (human) | 1029 | Forward  Reverse | GGGTTTTCGTGGTTCACATCC  CTAGACGCTGGCTCCTCAGTA |
| P21  (human) | 1026 | Forward  Reverse | TGTCCGTCAGAACCCATGC  AAAGTCGAAGTTCCATCGCTC |
| P53  (human) | 7157 | Forward  Reverse | CAGCACATGACGGAGGTTGT  TCATCCAAATACTCCACACGC |
| CDKN1B  (human) | 1027 | Forward  Reverse | AACGTGCGAGTGTCTAACGG  CCCTCTAGGGGTTTGTGATTCT |
| LncRNA (mouse)  Gm49317-201 |  | Forward  Reverse | GGAGTCCTCAGACGGACAGAA  ATAATCAGCTTCCGTGTTGCC |
| ACTB  (mouse) | 11461 | Forward  Reverse | GTGACGTTGACATCCGTAAAGA  GCCGGACTCATCGTACTCC |
| GAPDH  (mouse) | 14433 | Forward  Reverse | AGGTCGGTGTGAACGGATTTG  GGGGTCGTTGATGGCAACA |
| Mmp3  (mouse) | 17392 | Forward  Reverse | GGCCTGGAACAGTCTTGGC  TGTCCATCGTTCATCATCGTCA |
| Mmp13  (mouse) | 17386 | Forward  Reverse | TGTTTGCAGAGCACTACTTGAA  CAGTCACCTCTAAGCCAAAGAAA |
| Sox9  (mouse) | 20682 | Forward  Reverse | AGTACCCGCATCTGCACAAC  ACGAAGGGTCTCTTCTCGCT |
| Aggrecan  (mouse) | 11595 | Forward  Reverse | GTGGAGCCGTGTTTCCAAG  AGATGCTGTTGACTCGAACCT |
| P16^INK4a^  (mouse) | 12578 | Forward  Reverse | CGCAGGTTCTTGGTCACTGT  TGTTCACGAAAGCCAGAGCG |
| P21  (mouse) | 12575 | Forward  Reverse | CCTGGTGATGTCCGACCTG  CCATGAGCGCATCGCAATC |
| P53  (mouse) | 22059 | Forward  Reverse | CCCCTGTCATCTTTTGTCCCT  AGCTGGCAGAATAGCTTATTGAG |
| CDKN1B  (mouse) | 12576 | Forward  Reverse | TCAAACGTGAGAGTGTCTAACG  CCGGGCCGAAGAGATTTCTG |

Supplementary Table4 Sequences of ASOs, shRNAs and probes

| Human LncRNA AC006064.4-201 ASO#1 | GTTGAAGTCAGAGGAGACCA |
| --- | --- |
| Human LncRNA AC006064.4-201 ASO#2 | GACTGAGTGTGGCAGGGACT |
| Human PTBP1 shRNA#1 | GCGTGAAGATCCTGTTCAA |
| Human PTBP1 shRNA#2 | GCCTCAACGTCAAGTACAA |
| Human CDKN1B shRNA#1 | GGTGGACCACGAAGAGTTA |
| Human CDKN1B shRNA#2 | GGAGCAATGCGCAGGAATA |
| Mouse LncRNA Gm49317-201 ASO#1 | CCCATTGGTAAAGTTCACCG |
| Mouse LncRNA Gm49317-201 ASO#2 | GCTTTTACAGAGTCAGCTGC |
| Mouse CDKN1B shRNA#1 | GCAAGTGGAATTTCGACTT |
| Mouse CDKN1B shRNA#2 | CCAGGCGGTGCCTTTAATT |
| Probes used for RNA pull-down assay | |
| Human LncRNA AC006064.4-201 (5bio) probe#1 | 5bio-ACCACTTTGTCAAGCTCATG |
| Human LncRNA AC006064.4-201 (5bio) probe#2 | 5bio-CCTTGTCATGTACCATCAAT |
| Human LncRNA AC006064.4-201 (5bio) probe#3 | 5bio-CCTCTGACTTCAACAGCGAC |
| Mouse LncRNA Gm49317-201 (5bio) probe#1 | 5bio-ACTCCTCAGCAGCTTAGAGT |
| Mouse LncRNA Gm49317-201 (5bio) probe#2 | 5bio-TCTGGGTGTGAGTAATGGCA |
| Mouse LncRNA Gm49317-201 (5bio) probe#3 | 5bio-GAAGGAAGTCTTGGTAACCA |
| Probes used for FISH assay | |
| Human LncRNA AC006064.4-201 probe | 5‘GGTCTCCTCTGACTTCAACAGCGACACCCACTC-3’ |
| Mouse LncRNA Gm49317-201 probe | 5‘-GTGCGGTGAACTTTACCAATGGGATTT-3’ |

Supplementary Table5 Antibodies used in this study

| Mmp3 (western blot) | abcam (ab52915) |
| --- | --- |
| Mmp13 (western blot) | abcam (ab39012) |
| Sox9 (western blot) | abcam (ab185230) |
| Aggrecan (western blot) | abcam (ab3778) |
| P21 (western blot) | abcam (ab109520) |
| P53 (western blot) | abcam (ab26) |
| CDKN1B (western blot) | abcam (ab32034) |
| YBX3 (western blot) | Affinity (DF14571) |
| APOBEC3C (western blot) | abcam (ab181356) |
| PTBP1 (western blot) | abcam (ab133734) |
| LIN28A (western blot) | abcam (ab279647) |
| IMPDH1 (western blot) | Proteintech (22092-1-AP) |
| P16^INK4a^ (IF) | abcam (ab51243) |
| Col2a1 (IF) | abcam (ab34712) |
| CDKN1B (IF) | Proteintech (25614-1-AP) |
| PTBP1 (IF) | abcam (ab133734) |
